# Future Directions in Hypercalcemic and Normocalcemic Primary Hyperparathyroidism: FRAXplus for 10-Year Fracture Risk Assessment (A Retrospective Study)

**DOI:** 10.3390/life16060932

**Published:** 2026-06-01

**Authors:** Ana-Maria Gheorghe, Oana-Claudia Sima, Mihai Costachescu, Nina Ionovici, Mara Carsote

**Affiliations:** 1 PhD Doctoral School, “Carol Davila” University of Medicine and Pharmacy, 020021 Bucharest, Romania; ana-maria.gheorghe@drd.umfcd.ro (A.-M.G.); carsote_m@hotmail.com (M.C.); 2 Department of Endocrinology V, “C.I. Parhon” National Institute of Endocrinology, 011863 Bucharest, Romania; oana-claudia.sima@drd.umfcd.ro; 3 Department of Radiology and Medical Imaging, “Dr. Carol Davila” Central Military University Emergency Hospital, 010825 Bucharest, Romania; mihai.costachescu@drd.umfcd.ro; 4 Occupational Medicine Department, University of Medicine and Pharmacy of Craiova, 200349 Craiova, Romania; 5 Department of Endocrinology, “Carol Davila” University of Medicine and Pharmacy, 020021 Bucharest, Romania

**Keywords:** parathyroid, parathormone, calcium, osteoporosis, fracture, retrospective

## Abstract

**Background:** Osteoporosis/osteoporotic fractures are identified in both hypercalcemic (HC-HPT) and normocalcemic variant (NC-HPT) of primary hyperparathyroidism (HPT) at various rates. **Objective:** Noting the need of modern society to easily assess the osteoporotic fracture risk amid the diagnosis of HPT, we aimed to address this gap by analyzing the 10-year fracture risk assessment based on traditional FRAX (Fracture Risk Assessment Tool) model in comparison to the novel algorithm (FRAXplus), according to the adjustment for the presence of HPT, as well as for the use of lumbar bone mineral density (BMD) in menopausal women with HPT versus controls (non-HPT), respectively, between HC-HPT versus NC-HPT. **Methods:** For each patient, the latest algorithms of FRAX and FRAXplus provided the 10-year fracture risk for major osteoporotic fractures (MOF) and for hip fracture (HF) amid a single-center, retrospective, real-life study. **Results:** In total, 131 subjects were included: 51.15% had HPT (64.18% of them had HC-HPT) versus age-, menopause duration-, and body mass index-matched (HPT-free) controls. As a result, 10-year fracture risk for MOF and HF was statistically significantly higher in HPT versus controls only for the calculation with femoral neck BMD. FRAXplus showed that for both estimations (MOF and HF) with introduction of lumbar BMD remained higher than controls (4.55% vs. 3.7%, *p* = 0.004, respectively, 1.05% vs. 0.5%, *p* = 0.002). In HPT group, 10-year fracture risk for MOF and HF were higher if adjustment for HPT was applied. The highest 10-year fracture risk for MOF was obtained for HPT adjustment with femoral neck BMD (5.9%) versus the estimation without using femoral neck BMD (5.25%, *p* = 0.001), respectively, versus the probability with adjustment for lumbar BMD (4.55%, *p* < 0.001). The same observation was for HF: 1.4% versus 1.2% (*p* = 0.028), respectively, versus 1.05% (*p* < 0.001). In HPT group, parathormone level positively correlated with 10-year hip fracture risk with HPT adjustment, without femoral neck BMD (r = 0.257, *p* = 0.049). Bone formation marker P1NP negatively correlated with 10-year fracture risk for MOF without femoral neck BMD (r = −0.416, *p* = 0.043), respectively, with the estimation including HPT adjustment without femoral neck BMD (r = −0.404, *p* = 0.05), and with the 10-year HF risk calculated without femoral neck BMD (r = −0.407, *p* = 0.049). **Conclusions:** To our best knowledge, this is the first study to address the use of FRAXplus in HPT. The similar values between FRAX-based probabilities without the use of femoral neck BMD in HPT versus non-HPT controls suggested that this traditional estimation might not be so useful in HPT population, thus the need for novel models (HPT adjustment). HPT adjustment (FRAXplus) provided a higher MOF/HF risk versus non-adjustment (FRAX). All 10-year probabilities based on FRAX and FRAXplus models showed similar values in HC-HPT versus NC-HPT, which implies that current algorithms might not make a clear distinction between HPT subtypes, yet the statistically significant results within each of these subgroups sustain the FRAXplus application regardless of the variant.

## 1. Introduction

Primary hyperparathyroidism (HPT), a parathyroid tumor-related condition, associates an increasing incidence nowadays due to a wide access to calcium screening protocols and due to the introduction of surveillance strategies for hereditary syndromes that might involve HPT across lifespan [1,2,3,4]. On the other hand, parathyroid masses may be accidentally detected during routine imaging evaluation for unrelated purposes (e.g., anterior neck ultrasound, computed tomography, or magnetic resonance imaging), as, for instance, it has occurred during recent COVID-19 pandemic (and similarly reported for other endocrine incidentalomas, such as those originating from the adrenal and pituitary glands) [5,6,7,8,9].

Except for genetic forms, HPT more commonly affects middle-aged and menopausal women (two to three times more often than men) [10,11,12]. The classical panel of complications involves high calcium and high parathormone (PTH)-related ailments of the bone (osteoporosis, osteopenia, fractures, and bone pain) and kidneys (stones, nephrocalcinosis, and impairment of the renal function) [13,14,15,16,17]. Non-traditional landscape varies from pancreatitis to cognitive and psychiatric anomalies, cardiovascular outcomes, type 2 diabetes, etc., none of them representing a standalone indication of parathyroidectomy, according to the current level of statistical evidence [18,19,20,21].

The contemporary presentation has shifted to an asymptomatic type, which has an increasing incidence in developed countries. Nevertheless, this remains only the tip of the iceberg, and even in this instance, a heterogeneous rate of classical and non-classical complications has been found (a rate that cannot be entirely predicted from the first biological recognition of the condition) [22,23,24].

Moreover, normocalcemic HPT (NC-HPT), a newly described entity, involves a high PTH in addition to normal albumin-adjusted total and ionized serum calcium levels, based on two assays during a period of 3 to 6 months (in the absence of secondary/renal hyperparathyroidism and associated with a normal 24 h urinary calcium) [24]. Its real epidemiologic impact remains unknown; neither a clear management plan has been released so far, while the panel of surgery criteria currently overlaps with those for the hypercalcemic variant [24,25,26]. The clinical elements do not exclude the co-presence of traditional and non-traditional spectrum of HPT manifestations (e.g., osteoporosis/fragility fractures, renal lithiasis, etc.) [24,27,28,29]. The rate of progression to a hypercalcemic variant remains a matter of debate, noting that some authors suggested that this specific entity may represent an early stage of hypercalcemic HPT (HC-HPT) [24,30,31,32].

### Objective

Noting the need of modern society to easily assess the osteoporotic fracture risk amid the diagnosis of HPT (hypercalcemic or normocalcemic), we aimed to address this gap by analyzing the 10-year fracture risk assessment based on traditional FRAX (Fracture Risk Assessment Tool) model in comparison to the novel algorithm (FRAXplus), according to the adjustment for the presence of HPT, as well as for the use of lumbar bone mineral density (BMD) at dual-energy X-ray absorptiometry (DXA) in menopausal women with HPT versus controls (non-HPT), respectively, between HC-HPT versus NC-HPT.

## 2. Methods

### 2.1. Study Design

This was a retrospective, single-center, real-life study in a tertiary (university) center of endocrinology, conducted between December 2023 and December 2025. (Ethical approval: “C.I. Parhon” National Institute of Endocrinology, Bucharest, Romania—number 35 from 22 September 2025 and “Carol Davila” University of Medicine and Pharmacy, Bucharest, Romania—number 869 from 16 January 2026).

### 2.2. Study Population

We included women who were evaluated as inpatients with at least two hospitalizations during the mentioned time frame and who were diagnosed with HPT. They signed the informed consent to agree for the anonymous medical data collection according to the hospital protocol.

**Inclusion criteria:** females in menopause, age of 50 years or older, confirmed diagnosis of primary hyperparathyroidism (hypercalcemic or normocalcemic) based on total/ionized calcium and parathormone assays (at least two assays during a 3- to 6-month period of time).

They were assigned as hypercalcemic group (HC-HPT) or normocalcemic (NC-HPT) depending on the high or normal total/ionized serum calcium. Normal 24 h urinary calcium was mandatory for the normocalcemic group.

**Exclusion criteria:** history of parathyroidectomy, hypophysectomy, adrenalectomy, or bariatric surgery; suspected or confirmed hereditary HPT, pregnancy-related HPT or HPT with pediatric onset; renal hyperparathyroidism; abnormal kidney function; osteitis fibrosa cystica; lack of double testing with respect to the blood/urinary assays of the mineral metabolism; non-interpretable or not available DXA results (for lumbar spine, femoral neck, total hip, and third distal radius at non-dominant arm); suspected or confirmed endocrine (non-parathyroid) tumors and/or malignancies of any origin; osteomalacia and vitamin D deficiency (serum 25-hydroxyvitamin D < 30 ng/mL); acute infections of any type at the moment of blood/urinary assays; previous or current diagnosis of osteoporosis (other than the diagnosis that has been established at the moment of parathyroid disease confirmation) and exposure to specific medication against osteoporosis (e.g., bisphosphonates, denosumab, teriparatide, and romosozumab); suspected/confirmed parathyroid carcinoma based on the clinical, hormonal, imaging, and cytological/pathological evaluation; type 1 diabetes mellitus or endocrine (secondary) diabetes; rheumatoid arthritis; medical therapy that potentially involves an increase of the fracture risk and/or mineral metabolism disturbances at any point in life (glucocorticoids, aromatase inhibitors, insulin therapy, anticonvulsants, psychiatric medication, chemotherapy, immunotherapy, and anti-obesity drugs) or during latest 12 months (antibiotics, diuretics, and anti-ulcer drugs).

Of note, the subjects from HPT group were included regardless of the parathyroid tumors’ findings at imaging evaluation (except for a suspected/confirmed parathyroid malignancy) and irrespective if they later became parathyroidectomy candidates or were further conservatively managed.

**Figure 1:**
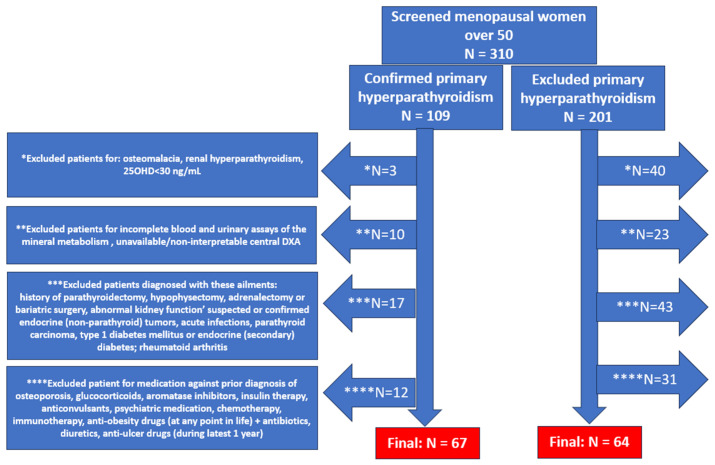
Flowchart diagram of patient’s inclusion/exclusion.

Control group were individuals without suspected/confirmed HPT who also checked all the other (non-HPT) inclusion criteria, as well as the previous mentioned exclusion criteria. Controls were consecutive patients who underwent hospitalization for various endocrine ailments (e.g., goiter or menopausal screening of bone status, specifically, osteoporosis based on central DXA, etc.), including suspected HPT, which was not confirmed. This is a hospital-based study (Figure 1).

### 2.3. Study Assessments

**Figure 2:**
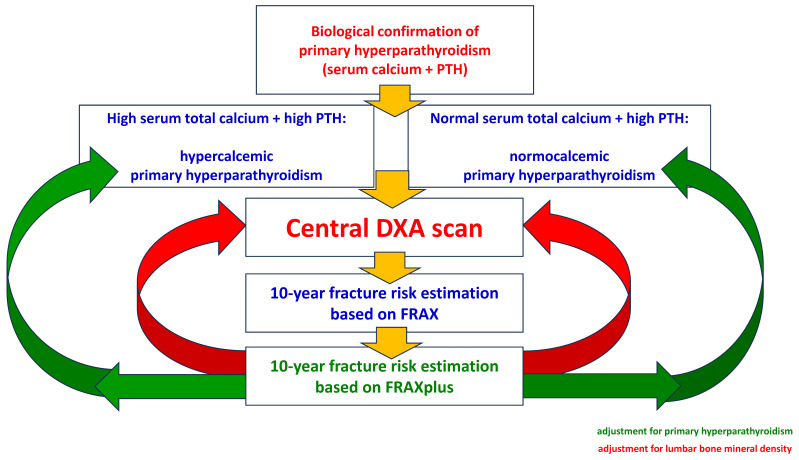
Study assessments.

The data collection via accessing the medical records/assessments during hospitalization included:

• demographic parameters (age, menopausal status).
• body mass index calculation (kg/m^2^).
• comorbidities’ profile [arterial hypertension, dyslipidemia of any type, impaired glucose profile (prediabetes and type 2 diabetes), and prior medication history (as presented above).
• blood/urinary biochemistry assays (serum total calcium, ionized calcium, phosphorus, magnesium (and calculation of total calcium/magnesium ratio), 24 h urinary calcium, serum creatinine, urea, uric acid, total cholesterol, triglycerides, fasting glycemia, and glycated hemoglobin A1c). Normocalcemic group required normal total and ionic serum calcium (formula: corrected calcium (mg/dL) = total calcium (mg/dL) + 0.8 × (4-serum albumin (g/dL)), normal serum creatinine and urea (and associated estimated glomerular filtration rate of >90 mL/min/1.73 m^2^), normal 25-hydroxyvitamin D, and exclusion of the mentioned drugs’ exposure that might interfere with the calcium levels.
• blood hormonal assays (25-hydroxyvitamin D, PTH).
• blood bone turnover markers [total alkaline phosphatase, osteocalcin, P1NP (procollagen 1 N-terminal propeptide) and CrossLaps].
• central DXA (GE Lunar Prodigy)—based L1-L4 lumbar spine, femoral neck and total hip, and third distal radius (non-dominant arm) BMD/T-score. WHO categories of osteoporosis, osteopenia, and normal DXA according to the lowest T-score were applied [33].
• lumbar DXA—based trabecular bone score (TBS).
• prevalent non-vertebral/vertebral fragility fractures according to the medical history of each patient and vertebral fractures screening based on thoracic-lumbar spine X-ray were included. (DXA scans, screening spine X-rays, and radiological records of the fractures were reanalyzed by three trained radiologists) (Figure 2).

### 2.4. The Application of FRAX and FRAXplus Tools

**Figure 3:**
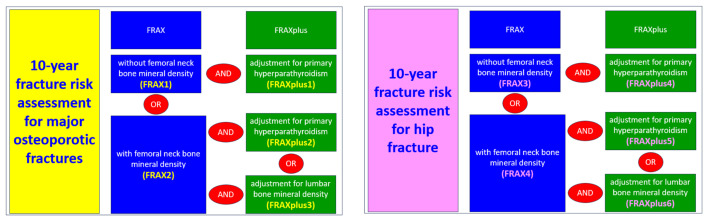
FRAX and FRAXplus models provided the 10-year fracture risk for major osteoporotic fractures (upper capture in yellow) and hip fracture (lower capture in pink): the figure introduces the designed terms for fracture probabilities according to the current study protocol.

For each patient, the latest algorithms of FRAX and FRAXplus (with adjustment for HPT, respectively, lumbar BMD) provided the 10-year fracture risk for major osteoporotic fractures (MOF) and 10-year fracture risk for hip fracture (HF) for the Romanian population [34]. The data collection included the conventional clinical risk factors (smoking, body mass index, etc.) in FRAX (Figure 3, Supplementary Materials).

### 2.5. Statistical Analysis

The statistical analysis was performed using IBM SPSS Statistics 29.0.2.0 (IBM Corp., Armonk, NY, USA), Microsoft Excel 16.106.1 (Microsoft Corp., Redmond, WA, USA), and GraphPad Prism 10.6.0 (GraphPad Software, Boston, MA, USA). Continuous variables were tested for normality using the Kolmogorov–Smirnov test and visual inspection of histograms. Data with normal distribution were expressed as mean ± standard deviation (SD), and non-normally distributed variables were presented as median and quartiles (Q1, Q3). Categorical variables were expressed as absolute frequencies and percentages. Between-group comparisons were performed using the independent samples Student’s t-test for normally distributed continuous variables and the Mann–Whitney U test for non-Gaussian parameters. Categorical variables were compared using Fisher’s exact test. Correlation analyses between Gaussian variables were performed using Pearson’s correlation coefficient for normally distributed variables and Spearman’s rank correlation coefficient otherwise. Receiver operating characteristic (ROC) curve analysis was conducted, and the area under the curve (AUC) was calculated with corresponding *p*-values. The optimal cutoff values were determined using the Youden index, and sensitivity and specificity were reported accordingly. All tests were two-tailed, and a *p*-value < 0.05 was considered statistically significant. Tests used for within-subject comparisons (Table 5, Table 8, Table 13, Table 14, Table 15 and Table 16) and for between-group comparisons (Table 1, Table 2, Table 3, Table 4, Table 9, Table 10, Table 11 and Table 12).

## 3. Results

### 3.1. Analysis of Patients with Primary Hyperparathyroidism Versus Control

A total of 131 patients were analyzed, with a mean age of 62.18 ± 8.51 years, 14.18 ± 8.78 years since menopause, and body mass index of 28.85 ± 4.68 kg/sqm. Additionally, 51.15% had HPT (group HPT), and 48.85% were age-, years since menopause-, and body mass index-matched controls (group control) (Table 1).

**Table 1:** Demographic and biochemical parameters in study group.

Parameter, Mean ± SD	Entire Group (N = 131, 100%)	Group HPT (N = 67, 51.15%)	Group Control (N = 64, 48.85%)	*p*-Value	Normal Range
Age (years)	62.18 ± 8.51	63.01 ± 8.45	61.31 ± 8.54	0.254	
Years since menopause	14.18 ± 8.78	14.52 ± 9.04	13.85 ± 8.59	0.682	
Body mass index (kg/sqm)	28.85 ± 4.68	28.36 ± 5.22	29.32 ± 4.09	0.260	
Impaired glucose profile, N (%)	59 (45.04)	30 (44.78)	29 (45.31)	0.951	
Dyslipidemia, N (%)	108 (82.44)	60 (89.55)	48 (75.00)	**0.016**	
Arterial hypertension, N (%)	87 (66.41)	48 (71.64)	39 (60.94)	0.195	
Serum creatinine (mg/dL)	0.79 ± 0.19	0.83 ± 0.22	0.75 ± 0.16	**0.022**	0.7–1.2
Serum urea (mg/dL)	39.51 ± 12.52	41.04 ± 14.82	38.10 ± 9.84	0.202	22–43
Uric acid (mg/dL)	5.76 ± 1.84	5.61 ± 1.69	5.89 ± 1.98	0.751	2.4–5.7
Fasting glycemia (mg/dL)	102.34 ± 16.74	101.03 ± 20.48	103.64 ± 11.94	0.396	80–115
Glycated hemoglobin A1c (%)	5.79 ± 0.66	5.80 ± 0.95	5.79 ± 0.37	0.914	4.8–5.9
Total cholesterol (mg/dL)	200.95 ± 45.03	199.32 ± 44.86	202.53 ± 45.50	0.690	0–200
Triglycerides (mg/dL)	124.53 ± 63.93	132.48 ± 71.95	117.07 ± 54.90	0.181	0–150

Abbreviations: HPT = primary hyperparathyroidism; SD = standard deviation.

Total serum calcium was statistically significantly increased in group HPT of 10.65 ± 0.96 mg/dL in comparison to group control of 9.45 ± 0.44 mg/dL (*p* < 0.001), as well as ionized serum calcium of 4.61 ± 0.47 mg/dL versus 4.03 ± 0.24 mg/dL (*p* < 0.001), but, also, PTH (123.68 ± 75.04 pg/mL vs. 38.25 ± 9.95 pg/mL, *p* < 0.001). Calcium/magnesium ratio was increased in group HPT of 5.47 ± 0.88 versus 4.74 ± 0.62 in group control (*p* < 0.001) (Table 2).

**Table 2:** Mineral metabolism assays and bone turnover markers of the entire group, group HPT, and group control.

Parameter, Mean ± SD	Entire Group (N = 131, 100%)	Group HPT (N = 67, 51.15%)	Group Control (N = 64, 48.85%)	*p*-Value	Normal Range
Total serum calcium (mg/dL)	10.06 ± 0.96	10.65 ± 0.96	9.45 ± 0.44	**<0.001**	8.4–10.2
Ionized serum calcium (mg/dL)	4.36 ± 0.48	4.61 ± 0.47	4.03 ± 0.24	**<0.001**	3.9–4.9
Total proteins (g/dL)	7.38 ± 0.49	7.32 ± 0.52	7.43 ± 0.46	0.246	6.5–8.7
Serum phosphorus (mg/dL)	3.35 ± 0.62	3.01 ± 0.58	3.67 ± 0.48	**<0.001**	2.3–4.7
Serum magnesium (mg/dL)	2.01 ± 0.22	1.99 ± 0.24	2.02 ± 0.21	0.433	1.6–2.6
Calcium/magnesium ratio	5.09 ± 0.83	5.47 ± 0.88	4.74 ± 0.62	**<0.001**	
25-hydroxyvitamin D (ng/mL)	30.51 ± 1.93	30.75 ± 0.11	32.91 ± 1.81	0.511	30–100
Parathormone (pg/mL)	81.62 ± 68.73	123.68 ± 75.04	38.25 ± 9.95	**<0.001**	15–65
Alkaline phosphatase (U/L), median (Q1, Q3)	80.20 (65.40, 103.00)	90.00 (73.00, 122.00)	74.90 (61.85, 91.68)	**0.005**	35–104
Osteocalcin (ng/mL), median (Q1, Q3)	26.35 (20.25, 36.04)	35.69 (23.63, 44.07)	23.24 (17.91, 28.45)	**<0.001**	15–46
P1NP (ng/mL), median (Q1, Q3)	60.74 (49.09, 78.26)	67.15 (46.52, 93.85)	60.25 (49.21, 75.55)	0.624	20.25–76.31
CrossLaps (ng/mL), median (Q1, Q3)	0.49 (0.33, 0.68)	0.67 (0.43, 0.78)	0.41 (0.32, 0.55)	**<0.001**	0.33–0.782

Abbreviations: HPT = primary hyperparathyroidism; Q1 = first quartile; Q3 = third quartile; SD = standard deviation.

**Figure 4:**
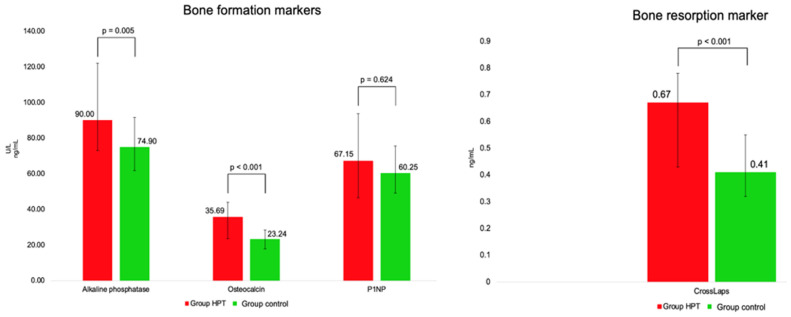
Bar charts with error bars showing mean and standard deviation of bone formation markers and bone resorption markers in group HPT versus group control.

Osteocalcin was higher in group HPT than group control (*p* < 0.001), as well as CrossLaps (*p* < 0.001) (Figure 4).

BMD and T-score at each central DXA site were statistically significantly lower in group HPT compared to group control (*p* < 0.001 for each) and TBS (1.239 ± 0.127 vs. 1.351 ± 0.104, *p* = 0.004). Group HPT had statistically significantly higher frequency of osteoporosis (*p* < 0.001) and lower rate of normal DXA (*p* < 0.001) (Table 3).

**Table 3:** DXA assessment of BMD, T-score, and TBS in the entire group and group HPT versus group control.

Parameter, Mean ± SD	Entire Group (N = 131, 100%)	Group HPT (N = 67, 51.15%)	Group Control (N = 64, 48.85%)	*p*-Value	Normal Range
Lumbar BMD (g/cm^2^)	0.994 ± 0.173	0.921 ± 0.159	1.067 ± 0.156	**<0.001**	
Lumbar T-score (SD)	−1.46 ± 1.41	−2.04 ± 1.35	−0.82 ± 1.20	**<0.001**	>−1
Femoral neck BMD (g/cm^2^)	0.828 ± 0.125	0.770 ± 0.106	0.881 ± 0.118	**<0.001**	
Femoral neck T-score (SD)	−1.31 ± 0.98	−1.80 ± 0.73	−0.83 ± 0.96	**<0.001**	>−1
Total hip BMD (g/cm^2^)	0.885 ± 0.217	0.783 ± 0.272	0.962 ± 0.120	**<0.001**	
Total hip T-score (SD)	−0.85 ± 1.16	−1.52 ± 0.99	−0.33 ± 1.02	**<0.001**	>−1
Third distal radius BMD (g/cm^2^)	0.693 ± 0.214	0.582 ± 0.231	0.823 ± 0.080	**<0.001**	
Third distal radius T-score (SD)	−1.49 ± 1.52	−2.21 ± 1.53	−0.57 ± 0.89	**<0.001**	>−1
TBS	1.331 ± 0.116	1.239 ± 0.127	1.351 ± 0.104	**0.004**	>1.350
Osteoporosis, N (%)	38 (29.01)	34 (50.75)	4 (6.25)	**<0.001**	
Osteopenia, N (%)	56 (42.75)	27 (40.30)	29 (45.31)	0.293	
Normal DXA, N (%)	26 (19.85)	3 (4.48)	23 (35.94)	**<0.001**	
Prevalent fragility fractures, N (%)	22 (16.79)	13 (19.40)	9 (14.06)	0.414	

Abbreviations: BMD = bone mineral density; DXA = dual-energy X-ray absorptiometry; SD = standard deviation; TBS = trabecular bone score.

Group HPT had a statistically significantly higher 10-year fracture risk for MOF based on FRAX model with femoral neck BMD [FRAX2 (*p* = 0.017)] versus controls. Additionally, 10-year fracture risk for MOF with lumbar BMD adjustment was higher than that of controls [FRAXplus3 (*p* = 0.004)]. Similar findings were identified for the 10-year hip fracture risk estimation [FRAX4 (*p* = 0.002), FRAXplus6 (*p* = 0.002)] (Table 4).

**Table 4:** FRAX- and FRAXplus-based 10-year probabilities of major osteoporotic fracture and hip fracture within the entire group, group HPT, and group control.

Parameter, Median (Q1, Q3)	Entire Group (N = 131, 100%)	Group HPT (N = 67, 51.15%)	Group Control (N = 64, 48.85%)	*p*-Value
		**10-year probability of MOF**		
FRAX1 (%)	3.80 (2.60, 5.90)	3.90 (2.73, 6.98)	3.80 (2.40, 5.50)	0.239
FRAX2 (%)	4.25 (3.03, 6.65)	4.80 (3.40, 8.50)	3.80 (2.60, 5.10)	**0.017**
FRAXplus1 (%)		5.25 (3.38, 8.40)		
FRAXplus2 (%)		5.90 (4.25, 10.40)		
FRAXplus3 (%)	4.20 (3.00, 6.80)	4.55 (3.55, 8.63)	3.70 (2.60, 5.00)	**0.004**
		**10-year probability of HF**		
FRAX3 (%)	0.60 (0.30, 1.60)	0.75 (0.33, 2.08)	0.60 (0.30, 1.30)	0.232
FRAX4 (%)	0.70 (0.30, 1.90)	1.00 (0.40, 2.70)	0.50 (0.30, 1.00)	**0.002**
FRAXplus4 (%)		1.20 (0.50, 3.08)		
FRAXplus5 (%)		1.40 (0.60, 3.75)		
FRAXplus6 (%)	0.70 (0.30, 1.70)	1.05 (0.50, 2.85)	0.50 (0.20, 0.90)	**0.002**

Abbreviations: BMD = bone mineral density; MOF = major osteoporotic fracture; HF = hip fracture; FRAX1 = MOF without femoral neck BMD; FRAX2 = MOF with femoral neck BMD; FRAXplus1 = MOF without femoral neck BMD adjusted for HPT; FRAXplus2 = MOF with femoral neck BMD adjusted for HPT; FRAXplus3 = MOF adjusted for lumbar BMD; FRAX3 = HF without femoral neck BMD; FRAX4 = HF with femoral neck BMD; FRAXplus4 = HF without femoral neck BMD adjusted for HPT; FRAXplus5 = HF with femoral neck BMD adjusted for HPT; FRAXplus6 = HF adjusted for lumbar BMD; HPT = primary hyperparathyroidism; Q1 = first quartile; Q3 = third quartile (of note: all FRAX- and FRAXplus-based probabilities show 10-year fracture risk estimation).

### 3.2. Analysis Within the Group with Primary Hyperparathyroidism

Within group HPT, serum total calcium was statistically significantly positively correlated with CrossLaps (r = 0.355, *p* = 0.010) and negatively correlated with lumbar BMD (r = −0.346, *p* = 0.009), lumbar T-score (r = −0.308, *p* = 0.015), and third distal radius T-score (r = −0.377, *p* = 0.010). PTH showed a statistically significant positive correlation with CrossLaps (r = 0.331, *p* = 0.017) and negative correlations with third distal radius BMD (r = −0.400, *p* = 0.010) and third distal radius T-score (r = −0.398, *p* = 0.007). TBS was negatively correlated with PTH (r = −0.873, *p* < 0.001) (Table 5).

**Table 5:** Correlations among mineral metabolism assays, bone turnover markers, and DXA parameters within group HPT.

Parameter	Alkaline Phosphatase (U/L)	Osteocalcin (ng/mL)	P1NP (ng/mL)	CrossLaps (ng/mL)	Lumbar BMD (g/cm^2^)	Lumbar T-Score (SD)	Femoral Neck BMD (g/cm^2^)	Femoral Neck T-Score (SD)	Total Hip BMD (g/cm^2^)	Total Hip T-Score (SD)	Third Distal Radius BMD (g/cm^2^)	Third Distal Radius T-Score (SD)	TBS
**Total serum calcium (mg/dL)**	r = 0.147 *p* = 0.286	r = 0.200 *p* = 0.155	r = 0.014 *p* = 0.943	**r = 0.355** ***p *** **= 0.010**	**r = −0.346** ***p *** **= 0.009**	**r = −0.308** ***p *** **= 0.015**	r = 0.074 *p* = 0.604	r = −0.061 *p* = 0.659	r = 0.125 *p* = 0.435	r = −0.081 *p* = 0.606	r = −0.280 *p* = 0.072	**r = −0.377** ***p *** **= 0.010**	r = −0.398 *p* = 0.255
**Ionized serum calcium (mg/dL)**	r = 0.035 *p* = 0.818	r = 0.212 *p* = 0.178	r = −0.030 *p* = 0.887	**r = 0.457** ***p *** **= 0.002**	**r = −0.323** ***p *** **= 0.032**	**r = −0.351** ***p *** **= 0.016**	r = 0.069 *p* = 0.678	r = 0.030 *p* = 0.854	r = −0.158 *p* = 0.397	r = −0.160 *p* = 0.383	**r = −0.347** ***p *** **= 0.041**	**r = −0.419** ***p *** **= 0.010**	r = −0.518 *p* = 0.233
**Serum phosphorus (mg/dL)**	r = 0.001 *p* = 0.993	r = −0.101 *p* = 0.491	r = 0.064 *p* = 0.747	r = −0.217 *p* = 0.138	**r = 0.397** ***p *** **= 0.004**	**r = 0.314** ***p *** **= 0.019**	r = 0.053 *p* = 0.721	r = 0.172 *p* = 0.232	r = 0.001 *p* = 0.998	**r = 0.338** ***p *** **= 0.035**	**r = 0.326** ***p *** **= 0.043**	**r = 0.337** ***p *** **= 0.031**	r = −0.009 *p* = 0.982
**Serum magnesium (mg/dL)**	r = 0.231 *p* = 0.096	r = −0.003 *p* = 0.984	r = 0.197 *p* = 0.315	r = 0.077 *p* = 0.601	r = −0.154 *p* = 0.276	r = −0.045 *p* = 0.745	r = −0.066 *p* = 0.658	r = −0.070 *p* = 0.630	r = 0.174 *p* = 0.297	r = −0.197 *p* = 0.230	r = 0.087 *p* = 0.588	r = 0.004 *p* = 0.981	r = 0.483 *p* = 0.188
**Calcium/magnesium ratio**	r = −0.093 *p* = 0.510	r = 0.138 *p* = 0.337	r = −0.138 *p* = 0.484	r = 0.134 *p* = 0.359	r = −0.122 *p* = 0.387	r = −0.214 *p* = 0.117	r = 0.097 *p* = 0.516	r = −0.070 *p* = 0.630	r = 0.174 *p* = 0.297	r = 0.083 *p* = 0.614	r = −0.300 *p* = 0.056	r = −0.246 *p* = 0.111	r = −0.514 *p* = 0.157
**25-hydroxyvitamin D (ng/mL)**	r = 0.111 *p* = 0.442	r = −0.024 *p* = 0.871	r = 0.229 *p* = 0.260	r = 0.155 *p* = 0.292	r = 0.211 *p* = 0.150	**r = 0.271** ***p *** **= 0.050**	r = −0.021 *p* = 0.891	r = −0.094 *p* = 0.521	r = 0.227 *p* = 0.190	r = −0.024 *p* = 0.892	r = −0.008 *p* = 0.964	r = 0.080 *p* = 0.630	**r = 0.824** ***p *** **= 0.006**
**Parathormone (pg/mL)**	r = 0.255 *p* = 0.060	r = 0.265 *p* = 0.057	r = −0.056 *p* = 0.777	**r = 0.331** ***p *** **= 0.017**	r = −0.242 *p* = 0.075	r = −0.247 *p* = 0.055	r = −0.104 *p* = 0.474	r = −0.138 *p* = 0.321	r = −0.124 *p* = 0.446	r = −0.129 *p* = 0.415	**r = −0.400** ***p *** **= 0.010**	**r = −0.398** ***p *** **= 0.007**	**r = −0.873** ***p *** **< 0.001**

Abbreviations: BMD = bone mineral density; DXA = dual-energy X-ray absorptiometry; P1NP = procollagen 1 N-terminal propeptide; SD = standard deviation; TBS = trabecular bone score.

**Figure 5:**
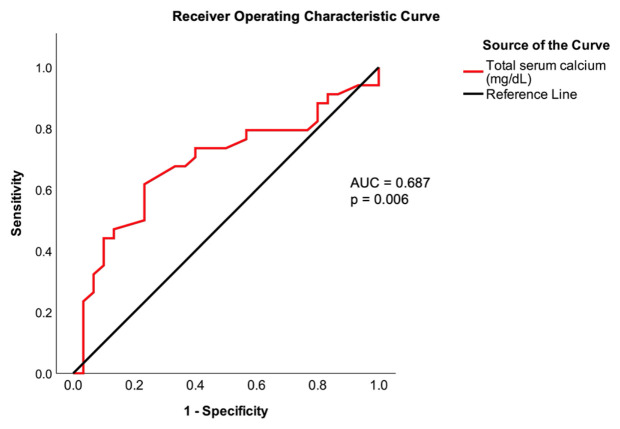
ROC curve of total serum calcium cutoff for predicting osteoporosis in group HPT. Abbreviations: AUC = area under the curve; HPT = primary hyperparathyroidism; ROC = receiver operating characteristic.

A ROC curve was plotted for determining sensitivity and 1-specificity of total serum calcium for predicting osteoporosis in patients from group HPT. AUC was statistically significant at 0.687 (*p* = 0.006), and the highest Youden index corresponded to a cutoff value of 10.75 mg/dL, with sensitivity of 61.80% and specificity of 76.70% (Table 6 and Figure 5).

**Table 6:** Sensitivity and specificity for the cutoff value of 10.75 mg/dL of total serum calcium for predicting osteoporosis among patients in group HPT.

Parameter	Cutoff Value for Predicting Osteoporosis (mg/dL)	AUC	Sensitivity (%)	Specificity (%)	Youden Index
**Total serum calcium**	10.75	0.687	61.80	76.70	0.384

Abbreviations: AUC = area under the curve; HPT = hyperparathyroidism.

**Figure 6:**
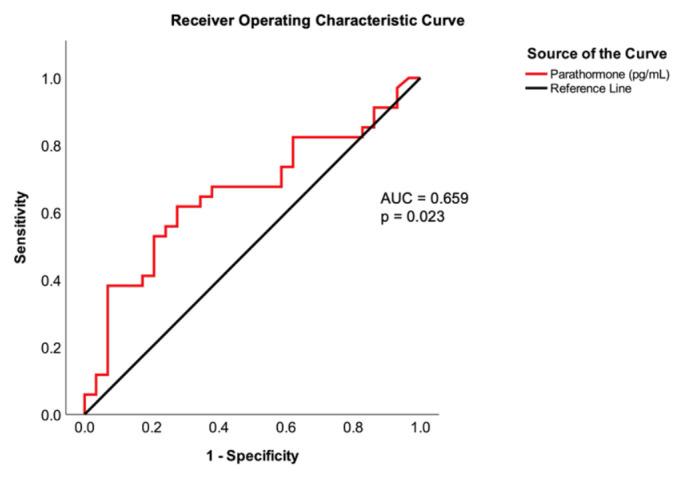
ROC curve of parathormone cutoff for predicting osteoporosis in group HPT. Abbreviations: AUC = area under the curve; HPT = primary hyperparathyroidism; ROC = receiver operating characteristic.

ROC analysis of PTH as potential parameter for predicting osteoporosis in group HPT yielded a statistically significant AUC of 0.659 (*p* = 0.023), with the highest Youden index related to a cutoff value of 100.28 pg/mL, with sensitivity of 61.80% and specificity of 72.40% (Table 7 and Figure 6).

**Figure 7:**
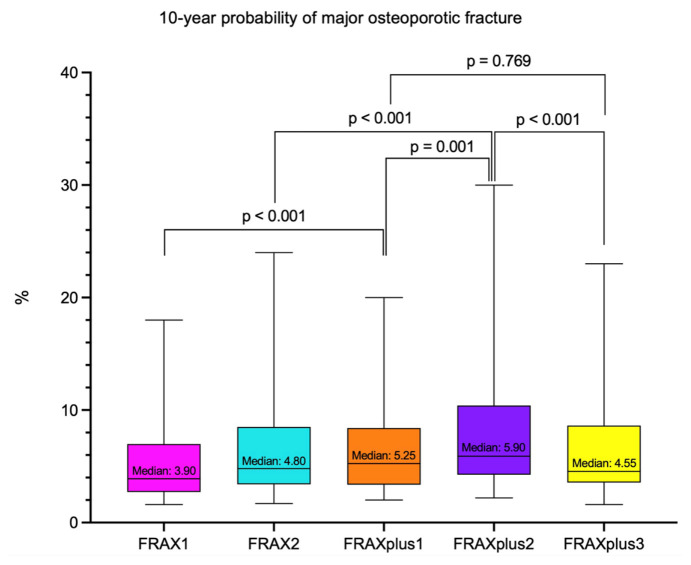
Box and whiskers plots showing median values for 10-year probabilities of major osteoporotic fracture estimated with FRAX and FRAXplus within group HPT. Abbreviations: BMD = bone mineral density; MOF = major osteoporotic fracture; FRAX1 = MOF without femoral neck BMD; FRAX2 = MOF with femoral neck BMD; FRAXplus1 = MOF without femoral neck BMD adjusted for HPT; FRAXplus2 = MOF with femoral neck BMD adjusted for HPT; FRAXplus3 = MOF adjusted for lumbar BMD; HPT = primary hyperparathyroidism (of note: all FRAX- and FRAXplus-based probabilities show 10-year fracture risk estimation).

Thus, 10-year fracture risk for MOF with HPT adjustment was elevated versus 10-year fracture risk without this HPT adjustment, regardless of whether the calculation did not include femoral neck BMD [FRAXplus1 vs. FRAX1 (*p* < 0.001)] or included it [FRAXplus2 vs. FRAX2 (*p* < 0.001)] (Figure 7).

**Figure 8:**
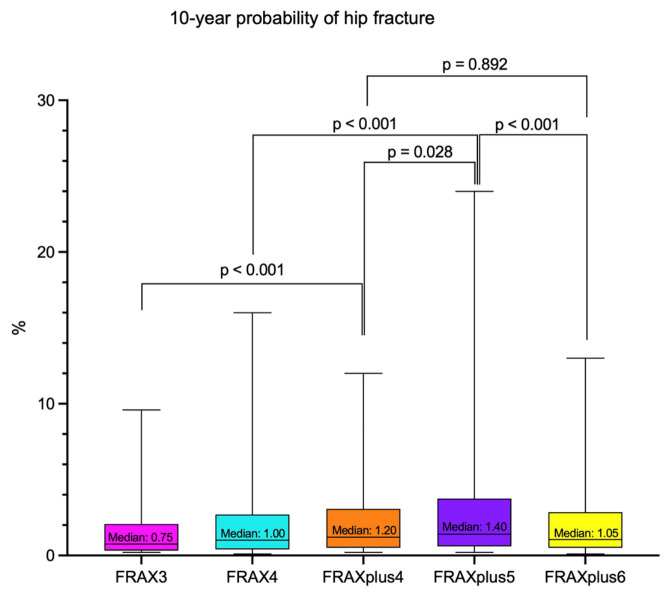
Box and whiskers plots showing median values for 10-year probabilities of hip fracture estimated with FRAX and FRAXplus within group HPT. Abbreviations: BMD = bone mineral density; HF = hip fracture; FRAX3 = HF without femoral neck BMD; FRAX4 = HF with femoral neck BMD; FRAXplus4 = HF without femoral neck BMD adjusted for HPT; FRAXplus5 = HF with femoral neck BMD adjusted for HPT; FRAXplus6 = HF adjusted for lumbar BMD; HPT = primary hyperparathyroidism (of note: all FRAX- and FRAXplus-based probabilities show 10-year fracture risk estimation).

Similarly, the 10-year hip fracture estimation showed that FRAXplus4 was statistically significantly higher in comparison to FRAX3 (*p* < 0.001). FRAXplus5 was higher versus FRAX4 (*p* < 0.001), respectively, versus FRAXplus4 (*p* = 0.028) and FRAXplus6 (*p* < 0.001). FRAXplus4 and FRAXplus6 were similar (*p* = 0.892) (Figure 8).

FRAXplus4 showed a statistically significant positive correlation with PTH (r = 0.257, *p* = 0.049). P1NP showed statistically significant negative correlations with FRAX1 (r = −0.416. *p* = 0.043), respectively, with FRAXplus1 (r = −0.404, *p* = 0.050) and FRAX3 (r = −0.407, *p* = 0.049). All FRAX- and FRAXplus-based parameters were statistically significantly and negatively correlated with femoral neck and total hip BMD and T-score. FRAX2, FRAXplus1, FRAXplus2, FRAXplus3, FRAX4, FRAXplus4, FRAXplus5, and FRAXplus6 were statistically significantly and negatively correlated with the third distal radius T-score (Table 8).

**Figure 9:**
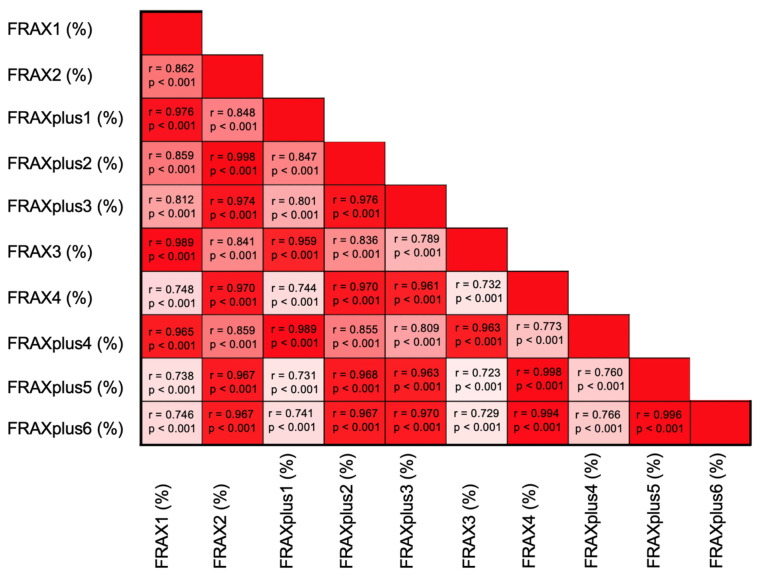
Correlation matrix showing correlation coefficients among FRAX- and FRAXplus-based 10-year probabilities of major osteoporotic fractures and hip fracture within group HPT. Abbreviations: BMD = bone mineral density; DXA = dual-energy X-ray absorptiometry; MOF = major osteoporotic fracture; HF = hip fracture; FRAX1 = MOF without femoral neck BMD; FRAX2 = MOF with femoral neck BMD; FRAXplus1 = MOF without femoral neck BMD adjusted for HPT; FRAXplus2 = MOF with femoral neck BMD adjusted for HPT; FRAXplus3 = MOF adjusted for lumbar BMD; FRAX3 = HF without femoral neck BMD; FRAX4 = HF with femoral neck BMD; FRAXplus4 = HF without femoral neck BMD adjusted for HPT; FRAXplus5 = HF with femoral neck BMD adjusted for HPT; FRAXplus6 = HF adjusted for lumbar BMD; HPT = primary hyperparathyroidism (of note: all FRAX- and FRAXplus-based probabilities show 10-year fracture risk estimation).

Statistically significant and positive correlations were found among all FRAX- and FRAXplus-based parameters within HPT group (Figure 9).

**Table 8:** Correlation of FRAX- and FRAXplus-based probabilities of 10-year major osteoporotic fracture and hip fracture with mineral metabolism assays, bone turnover markers, and DXA assessment in group HPT.

Parameter	Total Serum Calcium (mg/dL)	Serum Magnesium (mg/dL)	Calcium/Magnesium Ratio (mg/dL)	25-Hydroxyvitamin D (ng/mL)	Parathormone (pg/mL)	Alkaline Phosphatase (U/L)	Osteocalcin (ng/mL)	P1NP (ng/mL)	CrossLaps (ng/mL)	Lumbar BMD (g/cm^2^)	Lumbar T-Score (SD)	Femoral Neck BMD (g/cm^2^)	Femoral Neck T-Score (SD)	Total Hip BMD (g/cm^2^)	Total Hip T-Score (SD)	Third Distal Radius BMD (g/cm^2^)	Third Distal Radius T-Score (SD)
FRAX1 (%)	r = −0.176 *p* = 0.177	r = 0.152 *p* = 0.281	r = −0.264 *p* = 0.059	r = 0.083 *p* = 0.554	r = 0.201 *p* = 0.127	r = −0.171 *p* = 0.239	r = −0.239 *p* = 0.110	**r = −0.416** ***p *** **= 0.043**	r = −0.191 *p* = 0.203	r = −0.101 *p* = 0.483	r = −0.065 *p* = 0.636	**r = −0.461** ***p *** **= 0.001**	**r = −0.487** ***p *** **< 0.001**	**r = −0.423** ***p *** **= 0.011**	**r = −0.389** ***p *** **= 0.017**	r = −0.096 *p* = 0.577	r = −0.301 *p* = 0.063
FRAX2 (%)	r = −0.261 *p* = 0.083	r = 0.049 *p* = 0.762	r = −0.226 *p* = 0.156	r = 0.047 *p* = 0.775	r = 0.069 *p* = 0.655	r = −0.119 *p* = 0.230	r = −0.216 *p* = 0.200	r = −0.294 *p* = 0.197	r = −0.123 *p* = 0.474	r = −0.267 *p* = 0.080	r = −0.244 *p* = 0.111	**r = −0.817** ***p *** **< 0.001**	**r = −0.806** ***p *** **< 0.001**	**r = −0.651** ***p *** **< 0.001**	**r = −0.651** ***p *** **< 0.001**	r = −0.286 *p* = 0.119	**r = −0.492** ***p *** **= 0.005**
FRAXplus1 (%)	r = −0.144 *p* = 0.272	r = 0.175 *p* = 0.216	r = −0.233 *p* = 0.096	r = 0.097 *p* = 0.490	r = 0.251 *p* = 0.055	r = −0.139 *p* = 0.349	r = −0.237 *p* = 0.113	**r = −0.404** ***p *** **= 0.050**	r = −0.195 *p* = 0.194	r = −0.099 *p* = 0.494	r = −0.069 *p* = 0.619	**r = −0.463** ***p *** **= 0.001**	**r = −0.496** ***p *** **< 0.001**	**r = −0.392** ***p *** **= 0.020**	**r = −0.355** ***p *** **= 0.031**	r = −0.126 *p* = 0.464	**r = −0.347** ***p *** **= 0.030**
FRAXplus2 (%)	r = −0.257 *p* = 0.089	r = 0.058 *p* = 0.720	r = −0.228 *p* = 0.152	r = 0.048 *p* = 0.768	r = 0.076 *p* = 0.623	r = −0.194 *p* = 0.243	r = −0.212 *p* = 0.208	r = −0.296 *p* = 0.193	r = −0.123 *p* = 0.475	r = −0.269 *p* = 0.078	r = −0.246 *p* = 0.107	**r = −0.821** ***p *** **< 0.001**	**r = −0.808** ***p *** **< 0.001**	**r = −0.651** ***p *** **< 0.001**	**r = −0.649** ***p *** **< 0.001**	r = −0.293 *p* = 0.109	**r = −0.493** ***p *** **= 0.005**
FRAXplus3 (%)	r = −0.165 *p* = 0.286	r = 0.103 *p* = 0.523	r = −0.205 *p* = 0.198	r = −0.018 *p* = 0.914	r = 0.133 *p* = 0.394	r = −0.141 *p* = 0.398	r = −0.206 *p* = 0.222	r = −0.273 *p* = 0.231	r = −0.118 *p* = 0.491	**r = −0.403** ***p *** **= 0.007**	**r = −0.394** ***p *** **= 0.008**	**r = −0.840** ***p *** **< 0.001**	**r = −0.811** ***p *** **< 0.001**	**r = −0.670** ***p *** **< 0.001**	**r = −0.673** ***p *** **< 0.001**	r = −0.289 *p* = 0.115	**r = −0.485** ***p *** **= 0.006**
FRAX3 (%)	r = −0.173 *p* = 0.186	r = 0.160 *p* = 0.258	r = −0.267 *p* = 0.055	r = 0.051 *p* = 0.718	r = 0.215 *p* = 0.102	r = −0.155 *p* = 0.289	r = −0.225 *p* = 0.134	**r = −0.407** ***p *** **= 0.049**	r = −0.182 *p* = 0.227	r = −0.081 *p* = 0.576	r = −0.052 *p* = 0.706	**r = −0.433** ***p *** **= 0.003**	**r = −0.469** ***p *** **< 0.001**	**r = −0.392** ***p *** **= 0.020**	**r = −0.365** ***p *** **= 0.026**	r = −0.114 *p* = 0.508	r = −0.302 *p* = 0.062
FRAX4 (%)	r = −0.201 *p* = 0.185	r = 0.118 *p* = 0.461	r = −0.213 *p* = 0.180	r = 0.049 *p* = 0.766	r = 0.104 *p* = 0.501	r = −0.077 *p* = 0.646	r = −0.137 *p* = 0.419	r = −0.248 *p* = 0.279	r = −0.048 *p* = 0.782	**r = −0.349** ***p *** **= 0.020**	**r = −0.337** ***p *** **= 0.025**	**r = −0.903** ***p *** **< 0.001**	**r = −0.872** ***p *** **< 0.001**	**r = −0.766** ***p *** **< 0.001**	**r = −0.761** ***p *** **< 0.001**	**r = −0.364** ***p *** **= 0.044**	**r = −0.554** ***p *** **= 0.001**
FRAXplus4 (%)	r = −0.143 *p* = 0.275	r = 0.191 *p* = 0.175	r = −0.239 *p* = 0.087	r = 0.074 *p* = 0.599	**r = 0.257** ***p *** **= 0.049**	r = −0.112 *p* = 0.445	r = −0.222 *p* = 0.138	r = −0.382 *p* = 0.065	r = −0.171 *p* = 0.254	r = −0.115 *p* = 0.426	r = −0.085 *p* = 0.538	**r = −0.487** ***p *** **< 0.001**	**r = −0.514** ***p *** **< 0.001**	**r = −0.429** ***p *** **= 0.010**	**r = −0.387** ***p *** **= 0.018**	r = −0.169 *p* = 0.324	**r = −0.382** ***p *** **= 0.016**
FRAXplus5 (%)	r = −0.197 *p* = 0.194	r = 0.112 *p* = 0.485	r = −0.210 *p* = 0.188	r = 0.039 *p* = 0.810	r = 0.103 *p* = 0.508	r = −0.065 *p* = 0.700	r = −0.121 *p* = 0.476	r = −0.244 *p* = 0.286	r = −0.043 *p* = 0.802	**r = −0.366** ***p *** **= 0.015**	**r = −0.357** ***p *** **= 0.017**	**r = −0.905** ***p *** **< 0.001**	**r = −0.867** ***p *** **< 0.001**	**r = −0.773** ***p *** **< 0.001**	**r = −0.766** ***p *** **< 0.001**	**r = −0.355** ***p *** **= 0.050**	**r = −0.555** ***p *** **= 0.001**
FRAXplus6 (%)	r = −0.159 *p* = 0.303	r = 0.122 *p* = 0.448	r = −0.192 *p* = 0.229	r = 0.024 *p* = 0.884	r = 0.116 *p* = 0.458	r = −0.069 *p* = 0.679	r = −0.129 *p* = 0.446	r = −0.254 *p* = 0.267	r = −0.035 *p* = 0.840	**r = −0.407** ***p *** **= 0.006**	**r = −0.400** ***p *** **= 0.007**	**r = −0.909** ***p *** **< 0.001**	**r = −0.887** ***p *** **< 0.001**	**r = −0.782** ***p *** **< 0.001**	**r = −0.777** ***p *** **< 0.001**	r = −0.342 *p* = 0.060	**r = −0.564** ***p *** **< 0.001**

Abbreviations: BMD = bone mineral density; DXA = dual-energy X-ray absorptiometry; MOF = major osteoporotic fracture; HF = hip fracture; FRAX1 = MOF without femoral neck BMD; FRAX2 = MOF with femoral neck BMD; FRAXplus1 = MOF without femoral neck BMD adjusted for HPT; FRAXplus2 = MOF with femoral neck BMD adjusted for HPT; FRAXplus3 = MOF adjusted for lumbar BMD; FRAX3 = HF without femoral neck BMD; FRAX4 = HF with femoral neck BMD; FRAXplus4 = HF without femoral neck BMD adjusted for HPT; FRAXplus5 = HF with femoral neck BMD adjusted for HPT; FRAXplus6 = HF adjusted for lumbar BMD; HPT = primary hyperparathyroidism; P1NP = procollagen 1 N-terminal propeptide; SD = standard deviation (of note: all FRAX- and FRAXplus-based probabilities show 10-year fracture risk estimation).

### 3.3. Analysis of the Patients with Hypercalcemic Versus Normocalcemic Primary Hyperparathyroidism

In total, 64.18% of patients from group HPT had hypercalcemic type (group HC-HPT), and 35.82% had normocalcemic variant (group NC-HPT), with similar age, years since menopause, and body mass index (Table 9).

**Table 9:** Demographic parameters and biochemical assays of group HC-HPT versus group NC-HPT.

Parameter, Mean ± SD	Group HC-HPT (N = 43, 64.18%)	Group NC-HPT (N = 24, 35.82%)	*p*-Value
Age (years)	63.77 ± 8.48	61.67 ± 8.41	0.333
Years since menopause	15.11 ± 9.69	13.61 ± 8.06	0.539
Body mass index (kg/sqm)	29.25 ± 4.55	27.04 ± 5.95	0.109
Impaired glucose profile, N (%)	20 (46.51)	10 (41.67)	0.702
Dyslipidemia, N (%)	38 (88.37)	22 (91.67)	0.327
Arterial hypertension, N (%)	32 (74.42)	16 (66.67)	0.403
Serum creatinine (mg/dL)	0.81 ± 0.18	0.87 ± 0.27	0.335
Serum urea (mg/dL)	40.36 ± 13.70	42.37 ± 17.12	0.627
Uric acid (mg/dL	5.67 ± 1.40	4.59 ± 2.19	0.715
Fasting glycemia (mg/dL)	101.14 ± 23.90	100.81 ± 10.23	0.954
Glycated hemoglobin A1c (%)	5.94 ± 1.17	5.60 ± 0.39	0.256
Total cholesterol (mg/dL)	198.02 ± 46.70	202.04 ± 41.73	0.744
Triglycerides (mg/dL)	134.00 ± 73.83	128.95 ± 69.29	0.806

Abbreviations: HC-HPT = hypercalcemic primary hyperparathyroidism; NC-HPT = normocalcemic primary hyperparathyroidism; SD = standard deviation.

Total serum calcium and ionized serum calcium were statistically significantly increased in group HC-HPT versus group NC-HPT (*p* < 0.001 for each), as well as PTH (136.23 ± 87.21 vs. 101.72 ± 39.58 pg/mL, *p* = 0.032). Serum phosphorus was statistically significantly lower in group HC-HPT in comparison to group NC-HPT (*p* = 0.029). Group HC-HPT had higher calcium/magnesium ratio of 5.79 ± 0.86 than group NC-HPT of 4.84 ± 0.51 (*p* < 0.001). Bone turnover markers were similar (Table 10).

**Table 10:** Mineral metabolism, hormonal assays, and bone turnover markers in group HC-HPT and group NC-HPT.

Parameter, Mean ± SD	Group HC-HPT (N = 43, 64.18%)	Group NC-HPT (N = 24, 35.82%)	*p*-Value
Total serum calcium (mg/dL)	11.19 ± 0.70	9.66 ± 0.42	**<0.001**
Ionized serum calcium (mg/dL)	4.82 ± 0.41	4.21 ± 0.29	**<0.001**
Total proteins (g/dL)	7.33 ± 0.52	7.31 ± 0.52	0.905
Serum phosphorus (mg/dL)	2.78 ± 0.52	3.41 ± 0.46	**<0.001**
Serum magnesium (mg/dL)	1.98 ± 0.27	2.01 ± 0.17	0.540
Calcium/magnesium ratio	5.79 ± 0.86	4.84 ± 0.51	**<0.001**
25-hydroxyvitamin D (ng/mL)	30.58 ± 0.29	31.31 ± 0.24	0.290
Parathormone (pg/mL)	136.23 ± 87.21	101.72 ± 39.58	**0.032**
Alkaline phosphatase (U/L), median (Q1, Q3)	91.00 (71.00, 123.00)	80.50 (74.25, 106.75)	0.720
Osteocalcin (ng/mL), median (Q1, Q3)	35.69 (23.00, 48.41)	34.72 (24.59, 42.72)	0.758
P1NP (ng/mL), median (Q1, Q3)	62.11 (42.15, 115.23)	67.61 (48.15, 90.76)	0.890
CrossLaps (ng/mL), median (Q1, Q3)	0.68 (0.43, 0.99)	0.54 (0.41, 0.69)	0.141

Abbreviations: HC-HPT = hypercalcemic primary hyperparathyroidism; NC-HPT = normocalcemic primary hyperparathyroidism; P1NP = procollagen 1 N-terminal propeptide; Q1 = first quartile; Q3 = third quartile; SD = standard deviation.

Lumbar BMD and T-score were statistically significantly lower in group HC-HPT (*p* = 0.004, *p* = 0.010), respectively, total hip T-score (*p* = 0.034), third distal radius BMD, and T-score (*p* = 0.017, *p* = 0.005). Group HC-HPT had statistically significantly higher rate of osteoporosis (60.47%) versus group NC-HPT (33.33%) (*p* = 0.028) and lower rate of normal DXA (0.00% vs. 12.50%, *p* = 0.018). The frequency of prevalent fragility fractures was lower in group HC-HPT of 10.87% compared to group NC-HPT of 33.33% (*p* = 0.031) (Table 11).

**Table 11:** DXA assessment in group HC-HPT versus group NC-HPT.

Parameter, Mean ± SD	Group HC-HPT (N = 43, 64.18%)	Group NC-HPT (N = 24, 35.82%)	*p*-Value
Lumbar BMD (g/cm^2^)	0.875 ± 0.130	0.997 ± 0.175	**0.004**
Lumbar T-score (SD)	−2.36 ± 1.26	−1.45 ± 1.33	**0.010**
Femoral neck BMD (g/cm^2^)	0.771 ± 0.097	0.768 ± 0.123	0.925
Femoral neck T-score (SD)	−1.88 ± 0.74	−1.66 ± 0.72	0.282
Total hip BMD (g/cm^2^)	0.786 ± 0.117	0.779 ± 0.395	0.934
Total hip T-score (SD)	−1.79 ± 0.91	−1.14 ± 0.99	**0.034**
Third distal radius BMD (g/cm^2^)	0.519 ± 0.252	0.694 ± 0.132	**0.017**
Third distal radius T-score (SD)	−2.64 ± 1.42	−1.33 ± 1.40	**0.005**
TBS, mean ± SD	1.224 ± 0.143	1.274 ± 0.092	0.595
Osteoporosis, N (%)	26 (60.47)	8 (33.33)	**0.028**
Osteopenia, N (%)	15 (34.88)	12 (50.00)	0.226
Normal DXA, N (%)	0 (0.00)	3 (12.50)	**0.018**
Prevalent fragility fractures, N (%)	5 (10.87)	8 (33.33)	**0.031**

Abbreviations: BMD = bone mineral density; DXA = dual-energy X-ray absorptiometry; HC-HPT = hypercalcemic primary hyperparathyroidism; NC-HPT = normocalcemic primary hyperparathyroidism; SD = standard deviation; TBS = trabecular bone score.

FRAX- and FRAXplus-based parameters were similar between group HC-HPT and group NC-HPT (Table 12).

**Table 12:** 10-year probabilities of major osteoporotic fracture and hip fracture estimated with FRAX and FRAXplus in group HC-HPT and group NC-HPT.

Parameter, Median (Q1, Q3)	Group HC-HPT (N = 43, 64.18%)	Group NC-HPT (N = 24, 35.82%)	*p*-Value
**10-year probability of MOF**			
FRAX1 (%)	3.50 (2.83, 5.93)	5.15 (2.33, 8.15)	0.459
FRAX2 (%)	4.25 (3.18, 7.80)	5.30 (3.80, 9.10)	0.364
FRAXplus1 (%)	4.75 (3.60, 7.58)	6.35 (2.93, 10.70)	0.446
FRAXplus2 (%)	5.20 (3.98, 9.73)	6.40 (4.70, 11.00)	0.370
FRAXplus3 (%)	4.50 (3.38, 7.95)	5.05 (4.08, 8.88)	0.633
**10-year probability of HF**			
FRAX3 (%)	0.50 (0.40, 1.53)	1.15 (0.30, 2.68)	0.440
FRAX4 (%)	0.95 (0.40, 2.63)	1.10 (0.50, 3.60)	0.427
FRAXplus4 (%)	0.80 (0.50, 2.25)	1.55 (0.43, 3.83)	0.450
FRAXplus5 (%)	1.25 (0.58, 3.63)	1.50 (0.70, 5.20)	0.441
FRAXplus6 (%)	0.95 (0.48, 2.55)	1.10 (0.48, 3.58)	0.599

Abbreviations: BMD = bone mineral density; MOF = major osteoporotic fracture; HF = hip fracture; FRAX1 = MOF without femoral neck BMD; FRAX2 = MOF with femoral neck BMD; FRAXplus1 = MOF without femoral neck BMD adjusted for HPT; FRAXplus2 = MOF with femoral neck BMD adjusted for HPT; FRAXplus3 = MOF adjusted for lumbar BMD; FRAX3 = HF without femoral neck BMD; FRAX4 = HF with femoral neck BMD; FRAXplus4 = HF without femoral neck BMD adjusted for HPT; FRAXplus5 = HF with femoral neck BMD adjusted for HPT; FRAXplus6 = HF adjusted for lumbar BMD; HC-HPT = hypercalcemic primary hyperparathyroidism; NC-HPT = normocalcemic primary hyperparathyroidism; Q1 = first quartile; Q3 = third quartile (of note: all FRAX- and FRAXplus-based probabilities show 10-year fracture risk estimation).

### 3.4. Analysis Within the Group with Hypercalcemic Primary Hyperparathyroidism

Within group HC-HPT, a statistically significant positive correlation was found between total serum calcium and CrossLaps (r = 0.443, *p* = 0.010). Ionized serum calcium was positively correlated with CrossLaps (r = 0.577, *p* =0.002) and negatively correlated with third distal radius T-score (r = −0.465, *p* = 0.017). TBS negatively correlated with PTH (r = −0.857, *p* = 0.014) (Table 13).

**Table 13:** Correlations among mineral metabolism assays, bone turnover markers, and DXA parameters within group HC-HPT.

Parameter	Alkaline Phosphatase (U/L)	Osteocalcin (ng/mL)	P1NP (ng/mL)	CrossLaps (ng/mL)	Lumbar BMD (g/cm^2^)	Lumbar T-Score (SD)	Femoral Neck BMD (g/cm^2^)	Femoral Neck T-Score (SD)	Total Hip BMD (g/cm^2^)	Total Hip T-Score (SD)	Third Distal Radius BMD (g/cm^2^)	Third Distal Radius T-Score (SD)	TBS
Total serum calcium (mg/dL)	r = 0.212 *p* = 0.221	r = 0.288 *p* = 0.098	r = 0.323 *p* = 0.259	**r = 0.443** ***p *** **= 0.010**	r = −0.241 *p* = 0.164	r = −0.250 *p* = 0.119	r = −0.013 *p* = 0.946	r = −0.040 *p* = 0.823	r = 0.173 *p* = 0.430	r = 0.162 *p* = 0.439	r = −0.077 *p* = 0.702	r = −0.328 *p* = 0.072	r = −0.346 *p* = 0.448
Ionized serum calcium (mg/dL)	r = 0.205 *p* = 0.278	r = 0.292 *p* = 0.139	r = 0.259 *p* = 0.393	**r = 0.577** ***p *** **= 0.002**	r = −0.276 *p* = 0.164	r = −0.287 *p* = 0.124	r = −0.149 *p* = 0.488	r = −0.116 *p* = 0.572	r = −0.395 *p* = 0.117	r = −0.394 *p* = 0.105	r = −0.345 *p* = 0.099	**r = −0.465** ***p *** **= 0.017**	r = −0.678 *p* = 0.209
Serum phosphorus (mg/dL)	r = −0.222 *p* = 0.214	r = −0.344 *p* = 0.058	r = −0.060 *p* = 0.840	r = −0.255 *p* = 0.174	r = 0.306 *p* = 0.089	r = 0.158 *p* = 0.364	r = −0.022 *p* = 0.912	r = 0.074 *p* = 0.699	r = 0.376 *p* = 0.103	r = 0.381 *p* = 0.088	r = 0.007 *p* = 0.973	r = 0.122 *p* = 0.552	r = −0.450 *p* = 0.371
Serum magnesium (mg/dL)	r = 0.299 *p* = 0.086	r = 0.017 *p* = 0.928	r = 0.317 *p* = 0.269	r = 0.101 *p* = 0.587	r = −0.251 *p* = 0.159	r = −0.096 *p* = 0.578	r = −0.056 *p* = 0.771	r = 0.054 *p* = 0.774	r = −0.277 *p* = 0.224	r = −0.287 *p* = 0.196	r = −0.010 *p* = 0.963	r = −0.070 *p* = 0.723	r = 0.328 *p* = 0.526
Calcium/magnesium ratio	r = −0.153 *p* = 0.388	r = 0.122 *p* = 0.508	r = −0.196 *p* = 0.503	r = 0.057 *p* = 0.762	r = 0.111 *p* = 0.540	r = −0.048 *p* = 0.780	r = 0.032 *p* = 0.870	r = −0.101 *p* = 0.590	r = 0.349 *p* = 0.121	r = 0.345 *p* = 0.116	r = −0.115 *p* = 0.577	r = −0.107 *p* = 0.589	r = −0.409 *p* = 0.421
25-hydroxyvitamin D (ng/mL)	r = 0.068 *p* = 0.718	r = 0.061 *p* = 0.740	r = 0.500 *p* = 0.082	r = 0.289 *p* = 0.114	r = 0.330 *p* = 0.080	r = 0.325 *p* = 0.065	r = 0.121 *p* = 0.556	r = 0.081 *p* = 0.676	r = 0.007 *p* = 0.977	r = 0.027 *p* = 0.912	r = 0.013 *p* = 0.956	r = 0.156 *p* = 0.456	**r = 0.953** ***p *** **= 0.003**
Parathormone (pg/mL)	r = 0.292 *p* = 0.089	r = 0.282 *p* = 0.106	r = −0.143 *p* = 0.626	r = 0.243 *p* = 0.173	r = −0.228 *p* = 0.194	r = −0.215 *p* = 0.188	r = 0.022 *p* = 0.908	r = 0.020 *p* = 0.913	r = 0.135 *p* = 0.548	r = 0.169 *p* = 0.429	r = −0.262 *p* = 0.195	r = −0.248 *p* = 0.187	**r = −0.857** ***p *** **= 0.014**

Abbreviations: BMD = bone mineral density; DXA = dual-energy X-ray absorptiometry; HC-HPT = hypercalcemic primary hyperparathyroidism; P1NP = procollagen 1 N-terminal propeptide; SD = standard deviation; TBS = trabecular bone score.

**Figure 10:**
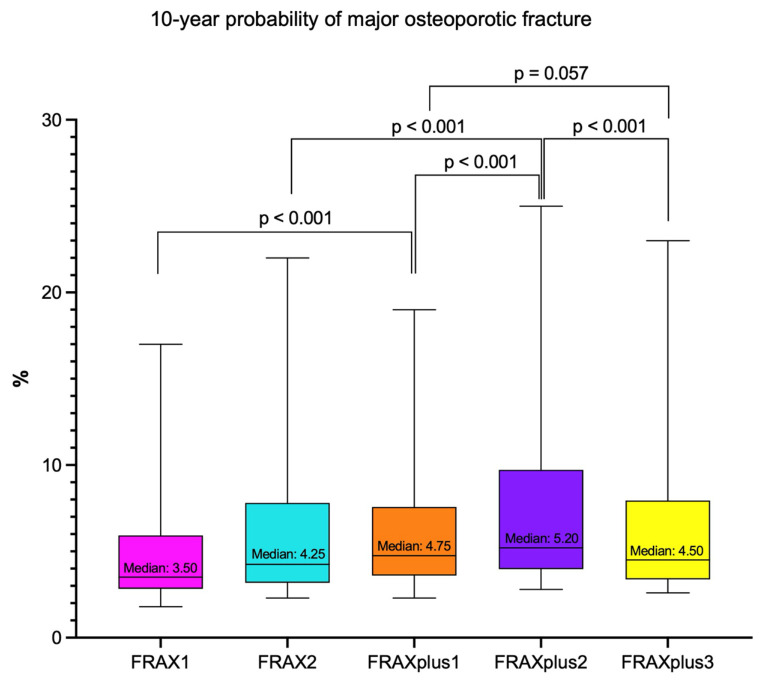
Box and whiskers plots showing median values for 10-year probabilities of major osteoporotic fracture estimated with FRAX and FRAXplus within group HC-HPT. Abbreviations: BMD = bone mineral density; MOF = major osteoporotic fracture; FRAX1 = MOF without femoral neck BMD; FRAX2 = MOF with femoral neck BMD; FRAXplus1 = MOF without femoral neck BMD adjusted for HPT; FRAXplus2 = MOF with femoral neck BMD adjusted for HPT; FRAXplus3 = MOF adjusted for lumbar BMD; HC-HPT = hypercalcemic primary hyperparathyroidism (of note: all FRAX- and FRAXplus-based probabilities show 10-year fracture risk estimation).

FRAXplus1 was statistically significantly higher compared to FRAX1 (*p* < 0.001). FRAXplus 2 was higher in comparison to FRAX2, FRAXplus1, and FRAXplus3 (*p* < 0.001 for each). FRAXplus1 and FRAXplus3 were similar between the groups (Figure 10).

**Figure 11:**
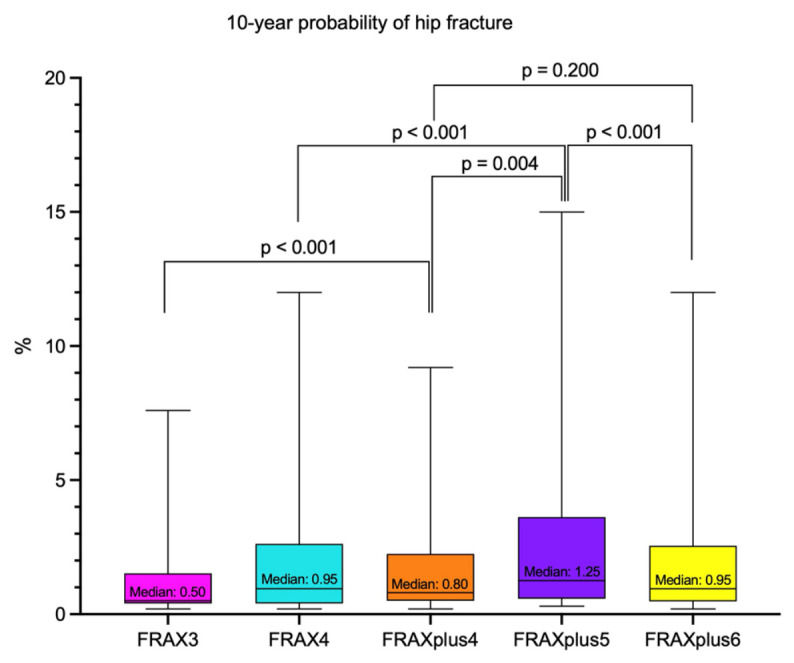
Box and whiskers plots showing median values for 10-year probabilities of hip fracture estimated with FRAX and FRAXplus within group HC-HPT. Abbreviations: BMD = bone mineral density; HF = hip fracture; FRAX3 = HF without femoral neck BMD; FRAX4 = HF with femoral neck BMD; FRAXplus4 = HF without femoral neck BMD adjusted for HPT; FRAXplus5 = HF with femoral neck BMD adjusted for HPT; FRAXplus6 = HF adjusted for lumbar BMD; HC-HPT = hypercalcemic primary hyperparathyroidism (of note: all FRAX- and FRAXplus-based probabilities show 10-year fracture risk estimation).

FRAXplus4 was statistically significantly higher compared to FRAX3 (*p* < 0.001). FRAXplus5 was higher in comparison to FRAX4 (*p* < 0.001), FRAXplus4 (*p* = 0.004), and FRAXplus6 (*p* < 0.001). FRAXplus4 and FRAXplus6 were similar between the groups (Figure 11).

All FRAX- and FRAXplus-based 10-year probabilities showed statistically significant and negative correlations with femoral neck BMD and femoral neck T-score. FRAXplus1 was negatively correlated with third distal radius T-score (r = −0.420, *p* = 0.041), as well as FRAXplus3 (r = −0.473, *p* = 0.047), FRAX4 (r = −0.596, *p* = 0.009), FRAXplus4 (r = −0.490, *p* = 0.015), FRAXplus5 (r = −0.605, *p* = 0.008), and FRAXplus6 (r = −0.577, *p* = 0.012) (Table 14).

**Table 14:** Correlation of FRAX- and FRAXplus-based probabilities of 10-year major osteoporotic fracture and hip fracture with mineral metabolism assays, bone turnover markers, and DXA assessment within group HC-HPT.

Parameter	Total Serum Calcium (mg/dL)	Serum Magnesium (mg/dL)	Calcium/Magnesium Ratio	25-Hydroxyvitamin D (ng/mL)	Parathormone (pg/mL)	Alkaline Phosphatase (U/L)	Osteocalcin (ng/mL)	P1NP (ng/mL)	CrossLaps (ng/mL)	Lumbar BMD (g/cm^2^)	Lumbar T-Score (SD)	Femoral Neck BMD (g/cm^2^)	Femoral Neck T-Score (SD)	Total Hip BMD (g/cm^2^)	Total Hip T-Score (SD)	Third Distal Radius BMD (g/cm^2^)	Third Distal Radius T-Score (SD)
FRAX1 (%)	r =−0.063 *p* = 0.714	r = −0.085 *p* = 0.643	r = 0.021 *p* = 0.909	r = −0.120 *p* = 0.521	r = −0.013 *p* = 0.943	r = −0.249 *p* = 0.192	r = −0.270 *p* = 0.164	r = −0.377 *p* = 0.283	r = −0.353 *p* = 0.071	r = −0.151 *p* = 0.435	r = −0.078 *p* = 0.666	**r = −0.529** ***p *** **= 0.005**	**r = −0.561** ***p *** **= 0.002**	r = −0.359 *p* = 0.157	r = −0.367 *p* = 0.123	r = −0.156 *p* = 0.499	r = −0.319 *p* = 0.128
FRAX2 (%)	r = −0.077 *p* = 0.709	r = −0.244 *p* = 0.250	r = 0.166 *p* = 0.439	r = −0.141 *p* = 0.531	r = −0.110 *p* = 0.600	r = −0.183 *p* = 0.415	r = −0.177 *p* = 0.442	r = −0.500 *p* = 0.170	r = −0.318 *p* = 0.171	r = −0.211 *p* = 0.300	r = −0.248 *p* = 0.221	**r = −0.793** ***p *** **< 0.001**	**r = −0.804** ***p *** **< 0.001**	r = −0.507 *p* = 0.064	r = −0.543 *p* = 0.045	r = −0.263 *p* = 0.292	r = −0.456 *p* = 0.057
FRAXplus1 (%)	r = 0.044 *p* = 0.798	r = −0.017 *p* = 0.924	r = 0.042 *p* = 0.819	r = −0.106 *p* = 0.572	r = 0.089 *p* = 0.612	r = −0.148 *p* = 0.445	r = −0.269 *p* = 0.167	r = −0.341 *p* = 0.334	r = −0.347 *p* = 0.076	r = −0.173 *p* = 0.369	r = −0.112 *p* = 0.535	**r = −0.536** ***p *** **= 0.005**	**r = −0.576** ***p *** **= 0.001**	r = −0.343 *p* = 0.178	r = −0.325 *p* = 0.174	r = −0.241 *p* = 0.293	**r = −0.420** ***p *** **= 0.041**
FRAXplus2 (%)	r = −0.068 *p* = 0.741	r = −0.227 *p* = 0.285	r = 0.155 *p* = 0.468	r = −0.138 *p* = 0.541	r = −0.099 *p* = 0.637	r = −0.178 *p* = 0.428	r = −0.177 *p* = 0.442	r = −0.500 *p* = 0.170	r = −0.318 *p* = 0.171	r = −0.220 *p* = 0.281	r = −0.253 *p* = 0.212	**r = −0.801** ***p *** **< 0.001**	**r = −0.809** ***p *** **< 0.001**	r = −0.504 *p* = 0.066	**r = −0.534** ***p *** **= 0.049**	r = −0.275 *p* = 0.270	r = −0.447 *p* = 0.063
FRAXplus3 (%)	r = 0.004 *p* = 0.985	r = −0.199 *p* = 0.351	r = 0.153 *p* = 0.476	r = −0.158 *p* = 0.483	r = −0.022 *p* = 0.916	r = −0.126 *p* = 0.577	r = −0.168 *p* = 0.467	r = −0.517 *p* = 0.154	r = −0.302 *p* = 0.195	r = 0.354 *p* = 0.076	**r = −0.392** ***p *** **= 0.048**	**r = −0.830** ***p *** **< 0.001**	**r = −0.832** ***p *** **< 0.001**	**r = −0.548** ***p *** **= 0.042**	**r = −0.585** ***p *** **= 0.028**	r = −0.275 *p* = 0.270	**r = −0.473** ***p *** **= 0.047**
FRAX3 (%)	r = −0.061 *p* = 0.723	r = −0.034 *p* = 0.854	r = −0.030 *p* = 0.869	r = −0.144 *p* = 0.441	r = 0.003 *p* = 0.984	r = −0.248 *p* = 0.194	r = −0.268 *p* = 0.167	r = −0.402 *p* = 0.249	r = −0.319 *p* = 0.104	r = −0.119 *p* = 0.540	r = −0.058 *p* = 0.748	**r = −0.505** ***p *** **= 0.009**	**r = −0.543** ***p *** **= 0.003**	r = −0.393 *p* = 0.119	r = −0.387 *p* = 0.102	r = −0.182 *p* = 0.430	r = −0.322 *p* = 0.125
FRAX4 (%)	r = 0.003 *p* = 0.987	r = −0.071 *p* = 0.742	r = 0.056 *p* = 0.796	r = −0.084 *p* = 0.711	r = −0.044 *p* = 0.835	r = −0.013 *p* = 0.954	r = −0.081 *p* = 0.728	r = −0.477 *p* = 0.194	r = −0.182 *p* = 0.444	r = −0.279 *p* = 0.168	r = −0.342 *p* = 0.087	**r = −0.903** ***p *** **< 0.001**	**r = −0.925** ***p *** **< 0.001**	**r = −0.756** ***p *** **= 0.002**	**r = −0.783** ***p *** **< 0.001**	r = −0.427 *p* = 0.078	**r = −0.596** ***p *** **= 0.009**
FRAXplus4 (%)	r = 0.023 *p* = 0.895	r = 0.051 *p* = 0.783	r = −0.016 *p* = 0.932	r = −0.152 *p* = 0.415	r = 0.089 *p* = 0.609	r = −0.106 *p* = 0.584	r = −0.239 *p* = 0.220	r = −0.377 *p* = 0.283	r = −0.310 *p* = 0.116	r = −0.162 *p* = 0.402	r = −0.121 *p* = 0.501	**r = −0.555** ***p *** **= 0.003**	**r = −0.599** ***p *** **< 0.001**	r = −0.422 *p* = 0.091	r = −0.379 *p* = 0.110	r = −0.312 *p* = 0.169	**r = −0.490** ***p *** **= 0.015**
FRAXplus5 (%)	r = 0.012 *p* = 0.952	r = −0.064 *p* = 0.765	r = 0.048 *p* = 0.823	r = −0.094 *p* = 0.678	r = −0.035 *p* = 0.867	r = 0.013 *p* = 0.954	r = −0.085 *p* = 0.714	r = −0.477 *p* = 0.194	r = −0.182 *p* = 0.442	r = −0.311 *p* = 0.122	r = −0.380 *p* = 0.055	**r = −0.897** ***p *** **< 0.001**	**r = −0.920** ***p *** **< 0.001**	**r = −0.767** ***p *** **= 0.001**	**r = −0.796** ***p *** **< 0.001**	r = −0.394 *p* = 0.106	**r = −0.605** ***p *** **= 0.008**
FRAXplus6 (%)	r = 0.025 *p* = 0.903	r = −0.091 *p* = 0.672	r = 0.079 *p* = 0.715	r = −0.113 *p* = 0.617	r = −0.044 *p* = 0.836	r = −0.014 *p* = 0.949	r = −0.082 *p* = 0.725	r = −0.477 *p* = 0.194	r = −0.180 *p* = 0.448	r = −0.356 *p* = 0.074	**r = −0.419** ***p *** **= 0.033**	**r = −0.900** ***p *** **< 0.001**	**r = −0.913** ***p *** **< 0.001**	**r = −0.773** ***p *** **= 0.001**	**r = −0.797** ***p *** **< 0.001**	r = −0.347 *p* = 0.158	**r = −0.577** ***p *** **= 0.012**

Abbreviations: BMD = bone mineral density; DXA = dual-energy X-ray absorptiometry; MOF = major osteoporotic fracture; HF = hip fracture; FRAX1 = MOF without femoral neck BMD; FRAX2 = MOF with femoral neck BMD; FRAXplus1 = MOF without femoral neck BMD adjusted for HPT; FRAXplus2 = MOF with femoral neck BMD adjusted for HPT; FRAXplus3 = MOF adjusted for lumbar BMD; FRAX3 = HF without femoral neck BMD; FRAX4 = HF with femoral neck BMD; FRAXplus4 = HF without femoral neck BMD adjusted for HPT; FRAXplus5 = HF with femoral neck BMD adjusted for HPT; FRAXplus6 = HF adjusted for lumbar BMD; HC-HPT = hypercalcemic primary hyperparathyroidism; P1NP = procollagen 1 N-terminal propeptide; SD = standard deviation (of note: all FRAX- and FRAXplus-based probabilities show 10-year fracture risk estimation).

**Figure 12:**
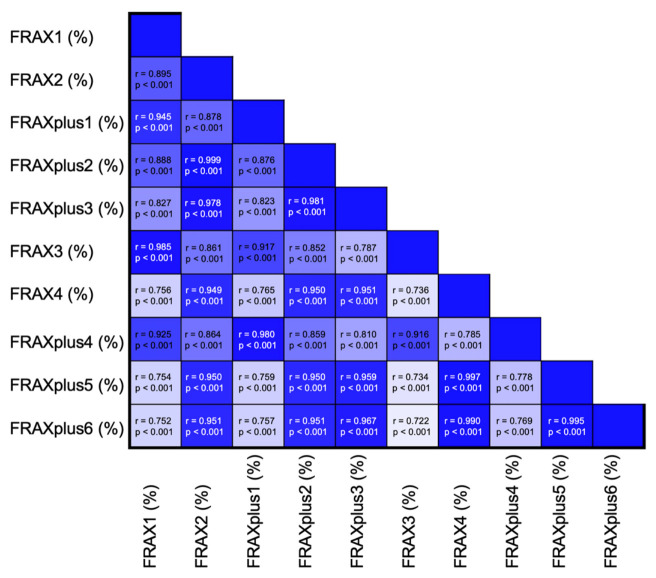
Correlation matrix showing correlation coefficients among FRAX- and FRAXplus-based probabilities of major osteoporotic fracture and hip fracture within group HC-HPT. Abbreviations: BMD = bone mineral density; DXA = dual-energy X-ray absorptiometry; MOF = major osteoporotic fracture; HF = hip fracture; FRAX1 = MOF without femoral neck BMD; FRAX2 = MOF with femoral neck BMD; FRAXplus1 = MOF without femoral neck BMD adjusted for HPT; FRAXplus2 = MOF with femoral neck BMD adjusted for HPT; FRAXplus3 = MOF adjusted for lumbar BMD; FRAX3 = HF without femoral neck BMD; FRAX4 = HF with femoral neck BMD; FRAXplus4 = HF without femoral neck BMD adjusted for HPT; FRAXplus5 = HF with femoral neck BMD adjusted for HPT; FRAXplus6 = HF adjusted for lumbar BMD; HC-HPT = hypercalcemic primary hyperparathyroidism (of note: all FRAX- and FRAXplus-based probabilities show 10-year fracture risk estimation).

Each FRAX-based estimation correlated with FRAXplus-based similar estimation within group HC-HPT (Figure 12).

### 3.5. Analysis Within the Group with Normocalcemic Primary Hyperparathyroidism

Ionized serum calcium was statistically significantly negatively correlated with alkaline phosphatase (r = −0.536, *p* = 0.039). Serum phosphorus was positively correlated with alkaline phosphatase (r = 0.558, *p* = 0.013), respectively, with osteocalcin (r = 0.666, *p* = 0.003). PTH showed a statistically significant and positive correlation with CrossLaps (r = 0.468, *p* = 0.043) and negative correlation with third distal radius T-score among patients in group NC-HPT (r = −0.587, *p* = 0.021) (Table 15).

**Table 15:** Correlations among mineral metabolism assays, bone turnover markers, and DXA parameters within group NC-HPT.

Parameter	Alkaline Phosphatase (U/L)	Osteocalcin (ng/mL)	P1NP (ng/mL)	CrossLaps (ng/mL)	Lumbar BMD (g/cm^2^)	Lumbar T-Score (SD)	Femoral Neck BMD (g/cm^2^)	Femoral Neck T-Score (SD)	Total Hip BMD (g/cm^2^)	Total Hip T-Score (SD)	Third Distal Radius BMD (g/cm^2^)	Third Distal Radius T-Score (SD)	TBS
Total serum calcium (mg/dL)	r = 0.070 *p* = 0.769	r = 0.246 *p* = 0.325	r = −0.256 *p* = 0.378	r = −0.103 *p* = 0.675	r = 0.231 *p* = 0.314	r = 0.312 *p* = 0.158	**r = 0.475** ***p *** **= 0.034**	**r = 0.435** ***p *** **= 0.049**	**r = 0.541** ***p *** **= 0.020**	**r = 0.564** ***p *** **= 0.015**	r = 0.436 *p* = 0.104	r = 0.464 *p* = 0.082	r = −0.500 *p* = 0.667
Ionized serum calcium (mg/dL)	**r = −0.536** ***p *** **= 0.039**	r = 0.045 *p* = 0.874	r = −0.543 *p* = 0.068	r = −0.323 *p* = 0.222	r = 0.032 *p* = 0.903	r = 0.092 *p* = 0.726	r = 0.178 *p* = 0.527	r = 0.119 *p* = 0.673	r = 0.496 *p* = 0.072	**r = 0.542** ***p *** **= 0.045**	r = −0.018 *p* = 0.957	r = −0.069 *p* = 0.840	r = −0.432 *p* = 0.638
Serum phosphorus (mg/dL)	**r = 0.558** ***p *** **= 0.013**	**r = 0.666** ***p *** **= 0.003**	r = 0.207 *p* = 0.478	r = 0.316 *p* = 0.201	r = 0.130 *p* = 0.586	r = 0.039 *p* = 0.869	r = 0.247 *p* = 0.294	r = 0.310 *p* = 0.183	r = −0.018 *p* = 0.945	r = −0.011 *p* = 0.966	r = 0.222 *p* = 0.426	r = −0.028 *p* = 0.922	r = 0.404 *p* = 0.511
Serum magnesium (mg/dL)	r = −0.066 *p* = 0.789	r = −0.088 *p* = 0.729	r = 0.024 *p* = 0.935	r = −0.119 *p* = 0.639	r = −0.270 *p* = 0.264	r = −0.259 *p* = 0.284	r = −0.384 *p* = 0.115	r = −0.335 *p* = 0.174	r = −0.318 *p* = 0.214	r = −0.281 *p* = 0.274	r = 0.157 *p* = 0.576	r = 0.020 *p* = 0.945	r = 0.367 *p* = 0.255
Calcium/magnesium ratio	r = 0.035 *p* = 0.887	r = 0.187 *p* = 0.458	r = −0.073 *p* = 0.805	r = 0.117 *p* = 0.645	r = 0.369 *p* = 0.120	r = 0.347 *p* = 0.146	**r = 0.517** ***p *** **= 0.028**	r = 0.464 *p* = 0.052	**r = 0.489** ***p *** **= 0.046**	r = 0.458 *p* = 0.064	r = 0.007 *p* = 0.980	r = 0.156 *p* = 0.580	r = −0.280 *p* = 0.318
25-hydroxyvitamin D (ng/mL)	r = 0.268 *p* = 0.267	r = −0.267 *p* = 0.300	r = −0.137 *p* = 0.655	r = 0.061 *p* = 0.815	r = 0.072 *p* = 0.770	r = 0.064 *p* = 0.788	r = −0.105 *p* = 0.668	r = −0.289 *p* = 0.217	r = −0.294 *p* = 0.252	r = −0.359 *p* = 0.157	r = −0.266 *p* = 0.358	r = −0.112 *p* = 0.702	r = −0.520 *p* = 0.684
Parathormone (pg/mL)	r =0.125 *p* = 0.600	r = 0.284 *p* = 0.254	r = 0.138 *p* = 0.637	**r = 0.468** ***p *** **= 0.043**	r = −0.031 *p* = 0.895	r = −0.175 *p* = 0.436	r = −0.167 *p* = 0.482	r = −0.108 *p* = 0.640	r = −0.390 *p* = 0.109	r = −0.412 *p* = 0.090	r = −0.089 *p* = 0.752	**r = −0.587** ***p *** **= 0.021**	r = −0.462 *p* = 0.550

Abbreviations: BMD = bone mineral density; DXA = dual-energy X-ray absorptiometry; NC-HPT = normocalcemic hyperparathyroidism; P1NP = procollagen 1 N-terminal propeptide; SD = standard deviation; TBS = trabecular bone score.

**Figure 13:**
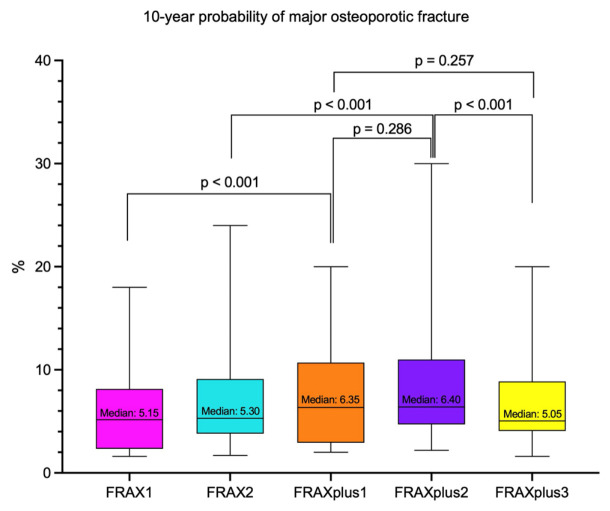
Box and whiskers plots showing median values for 10-year probabilities of major osteoporotic fracture estimated with FRAX and FRAXplus within group NC-HPT. Abbreviations: BMD = bone mineral density; MOF = major osteoporotic fracture; FRAX1 = MOF without femoral neck BMD; FRAX2 = MOF with femoral neck BMD; FRAXplus1 = MOF without femoral neck BMD adjusted for HPT; FRAXplus2 = MOF with femoral neck BMD adjusted for HPT; FRAXplus3 = MOF adjusted for lumbar BMD; NC-HPT = normocalcemic primary hyperparathyroidism (of note: all FRAX- and FRAXplus-based probabilities show 10-year fracture risk estimation).

FRAXplus1 was statistically significantly higher versus FRAX1 (*p* < 0.001). FRAXplus2 was statistically significantly higher compared to FRAX2 (*p* < 0.001), respectively, to FRAXplus3 (*p* < 0.001). FRAXplus1 was similar to FRAXplus2 and FRAXplus3 (Figure 13).

**Figure 14:**
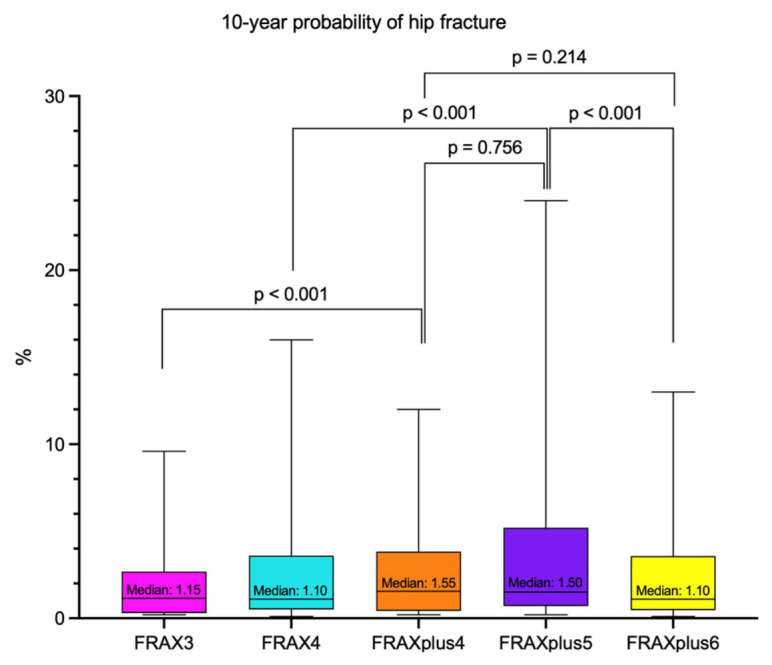
Box and whiskers plots showing median values for 10-year probabilities of hip fracture estimated with FRAX and FRAXplus within group NC-HPT. Abbreviations: BMD = bone mineral density; HF = hip fracture; FRAX3 = HF without femoral neck BMD; FRAX4 = HF with femoral neck BMD; FRAXplus4 = HF without femoral neck BMD adjusted for HPT; FRAXplus5 = HF with femoral neck BMD adjusted for HPT; FRAXplus6 = HF adjusted for lumbar BMD; NC-HPT = normocalcemic primary hyperparathyroidism (of note: all FRAX- and FRAXplus-based probabilities show 10-year fracture risk estimation).

FRAXplus4 was statistically significantly higher versus FRAX3 (*p* < 0.001). FRAXplus5 was higher than FRAX 4 (*p* < 0.001), respectively, than FRAXplus6 (*p* < 0.001). FRAXplus4 and FRAXplus5 were similar, as well as FRAXplus4 and FRAXplus6 (Figure 14).

Statistically significant and negative correlations were found between parathormone and FRAX1 (r = 0.524, *p* = 0.009), FRAXplus1 (r = 0.549, *p* = 0.005), FRAX3 (r = 0.483, *p* = 0.017), and FRAXplus4 (r = 0.519, *p* = 0.009). All FRAX and FRAXplus parameters were statistically significantly and negatively correlated with femoral neck BMD/T-score, total hip T-score, and third distal radius T-score (Table 16).

**Figure 15:**
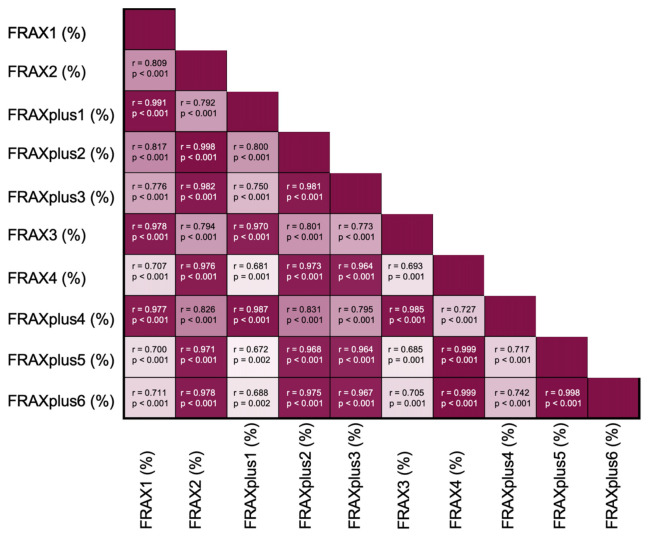
Correlation matrix showing correlation coefficients among FRAX- and FRAXplus-based probabilities of major osteoporotic fracture and hip fracture within group NC-HPT. Abbreviations: BMD = bone mineral density; DXA = dual-energy X-ray absorptiometry; MOF = major osteoporotic fracture; HF = hip fracture; FRAX1 = MOF without femoral neck BMD; FRAX2 = MOF with femoral neck BMD; FRAXplus1 = MOF without femoral neck BMD adjusted for HPT; FRAXplus2 = MOF with femoral neck BMD adjusted for HPT; FRAXplus3 = MOF adjusted for lumbar BMD; FRAX3 = HF without femoral neck BMD; FRAX4 = HF with femoral neck BMD; FRAXplus4 = HF without femoral neck BMD adjusted for HPT; FRAXplus5 = HF with femoral neck BMD adjusted for HPT; FRAXplus6 = HF adjusted for lumbar BMD; NC-HPT = normocalcemic primary hyperparathyroidism (of note: all FRAX- and FRAXplus-based probabilities show 10-year fracture risk estimation).

Pairwise analysis of correlations among FRAX and FRAXplus variables revealed statistically significant positive correlations between all probabilities in group NC-HPT (Figure 15).

**Table 16:** Correlation of FRAX- and FRAXplus-based probabilities of 10-year major osteoporotic fracture and hip fracture with mineral metabolism assays, bone turnover markers, and DXA assessment within group NC-HPT.

Parameter	Total Serum Calcium (mg/dL)	Serum Magnesium (mg/dL)	Calcium/Magnesium Ratio	25-Hydroxyvitamin D (ng/mL)	Parathormone (pg/mL)	Alkaline Phosphatase (U/L)	Osteocalcin (ng/mL)	P1NP (ng/mL)	CrossLaps (ng/mL)	Lumbar BMD (g/cm2)	Lumbar T-Score (SD)	Femoral Neck BMD (g/cm2)	Femoral Neck T-Score (SD)	Total Hip BMD (g/cm2)	Total Hip T-Score (SD)	Third Distal Radius BMD (g/cm^2^)	Third Distal Radius T-Score (SD)
FRAX1 (%)	r = −0.367 *p* = 0.078	**r = 0.473** ***p *** **= 0.035**	**r = −0.543** ***p *** **= 0.013**	**r = 0.445** ***p *** **= 0.038**	**r = 0.524** ***p *** **= 0.009**	r = 0.005 *p* = 0.982	r = −0.145 *p* = 0.567	r = −0.343 *p* = 0.230	r = 0.048 *p* = 0.844	r = −0.267 *p* = 0.242	r = −0.254 *p* = 0.255	**r = −0.499** ***p *** **= 0.025**	**r = −0.501** ***p *** **= 0.021**	**r = −0.557** ***p *** **= 0.016**	**r = −0.566** ***p *** **= 0.014**	r = −0.129 *p* = 0.647	**r = −0.516** ***p *** **= 0.049**
FRAX2 (%)	**r = −0.578** ***p *** **= 0.010**	r = 0.448 *p* = 0.072	**r = −0.581** ***p *** **= 0.014**	r = 0.199 *p* = 0.428	r = 0.323 *p* = 0.178	r = −0.182 *p* = 0.499	r = −0.262 *p* = 0.327	r = −0.140 *p* = 0.665	r = 0.121 *p* = 0.656	**r = −0.550** ***p *** **= 0.018**	**r = −0.566** ***p *** **= 0.014**	**r = −0.905** ***p *** **< 0.001**	**r = −0.865** ***p *** **< 0.001**	**r = −0.714** ***p *** **= 0.002**	**r = −0.715** ***p *** **= 0.002**	r = −0.346 *p* = 0.247	**r = −0.661** ***p *** **= 0.014**
FRAXplus1 (%)	r = −0.344 *p* = 0.100	r = 0.393 *p* = 0.086	**r = −0.467** ***p *** **= 0.038**	**r = 0.446** ***p *** **= 0.037**	**r = 0.549** ***p *** **= 0.005**	r = −0.055 *p* = 0.818	r = −0.140 *p* = 0.581	r = −0.350 *p* = 0.220	r = 0.035 *p* = 0.886	r = −0.233 *p* = 0.309	r = −0.220 *p* = 0.325	**r = −0.473** ***p *** **= 0.035**	**r = −0.479** ***p *** **= 0.028**	**r = −0.507** ***p *** **= 0.032**	**r = −0.517** ***p *** **= 0.028**	r = −0.152 *p* = 0.588	**r = −0.559** ***p *** **= 0.030**
FRAXplus2 (%)	**r = −0.575** ***p *** **= 0.010**	r = 0.456 *p* = 0.066	**r = −0.589** ***p *** **= 0.013**	r = 0.201 *p* = 0.423	r = 0.335 *p* = 0.161	r = −0.182 *p* = 0.499	r = −0.256 *p* = 0.338	r = −0.147 *p* = 0.648	r = 0.122 *p* = 0.652	**r = −0.543** ***p *** **= 0.020**	**r = −0.559** ***p *** **= 0.016**	**r = −0.899** ***p *** **< 0.001**	**r = −0.861** ***p *** **< 0.001**	**r = −0.714** ***p *** **= 0.002**	**r = −0.715** ***p *** **= 0.002**	r = −0.346 *p* = 0.247	**r = −0.661** ***p *** **= 0.014**
FRAXplus3 (%)	**r = −0.554** ***p *** **= 0.017**	r = 0.478 *p* = 0.052	**r = −0.599** ***p *** **= 0.011**	r = 0.207 *p* = 0.425	r = 0.311 *p* = 0.209	r = −0.167 *p* = 0.538	r = −0.303 *p* = 0.253	r = −0.175 *p* = 0.586	r = 0.046 *p* = 0.867	**r = −0.639** ***p *** **= 0.004**	**r = −0.665** ***p *** **= 0.003**	**r = −0.924** ***p *** **< 0.001**	**r = −0.815** ***p *** **< 0.001**	**r = −0.696** ***p *** **= 0.003**	**r = −0.698** ***p *** **= 0.003**	r = −0.328 *p* = 0.274	**r = −0.588** ***p *** **= 0.034**
FRAX3 (%)	r = −0.388 *p* = 0.061	**r = 0.469** ***p *** **= 0.037**	**r = −0.538** ***p *** **= 0.014**	r = 0.404 *p* = 0.062	**r = 0.483** ***p *** **= 0.017**	r = 0.054 *p* = 0.820	r = −0.122 *p* = 0.629	r = −0.311 *p* = 0.279	r = 0.042 *p* = 0.864	r = −0.238 *p* = 0.299	r = −0.238 *p* = 0.286	**r = −0.471** ***p *** **= 0.036**	**r = −0.485** ***p *** **= 0.026**	r = −0.459 *p* = 0.055	**r = −0.471** ***p *** **= 0.049**	r = −0.190 *p* = 0.498	**r = −0.558** ***p *** **= 0.031**
FRAX4 (%)	**r = −0.529** ***p *** **= 0.020**	r = 0.424 *p* = 0.090	**r = −0.544** ***p *** **= 0.024**	r = 0.154 *p* = 0.542	r = 0.247 *p* = 0.308	r = −0.178 *p* = 0.509	r = −0.263 *p* = 0.324	r = −0.112 *p* = 0.729	r = 0.132 *p* = 0.625	**r = −0.643** ***p *** **= 0.004**	**r = −0.661** ***p *** **= 0.003**	**r = −0.960** ***p *** **< 0.001**	**r = −0.857** ***p *** **< 0.001**	**r = −0.761** ***p *** **< 0.001**	**r = −0.756** ***p *** **< 0.001**	r = −0.344 *p* = 0.250	**r = −0.643** ***p *** **= 0.018**
FRAXplus4 (%)	r = −0.361 *p* = 0.083	r = 0.377 *p* = 0.101	**r = −0.452** ***p *** **= 0.046**	**r = 0.438** ***p *** **= 0.042**	**r = 0.519** ***p *** **= 0.009**	r = −0.038 *p* = 0.875	r = −0.125 *p* = 0.621	r = 0.282 *p* = 0.329	r = 0.069 *p* = 0.780	r = −0.251 *p* = 0.272	r = −0.245 *p* = 0.272	**r = −0.514** ***p *** **= 0.020**	**r = −0.514** ***p *** **= 0.017**	**r = −0.497** ***p *** **= 0.036**	**r = −0.510** ***p *** **= 0.031**	r = −0.220 *p* = 0.431	**r = −0.605** ***p *** **= 0.017**
FRAXplus5 (%)	**r = −0.518** ***p *** **= 0.023**	r = 0.427 *p* = 0.087	**r = −0.547** ***p *** **= 0.023**	r = 0.147 *p* = 0.562	r = 0.238 *p* = 0.327	r = −0.166 *p* = 0.538	r = −0.263 *p* = 0.324	r = −0.112 *p* = 0.729	r = 0.132 *p* = 0.625	**r = −0.645** ***p *** **= 0.004**	**r = −0.661** ***p *** **= 0.003**	**r = −0.964** ***p *** **< 0.001**	**r = −0.851** ***p *** **< 0.001**	**r = −0.764** ***p *** **< 0.001**	**r = −0.758** ***p *** **< 0.001**	r = −0.344 *p* = 0.250	**r = −0.643** ***p *** **= 0.018**
FRAXplus6 (%)	**r = −0.521** ***p *** **= 0.027**	r = 0.425 *p* = 0.089	**r = −0.544** ***p *** **= 0.024**	r = 0.166 *p* = 0.525	r = 0.269 *p* = 0.281	r = −0.172 *p* = 0.523	r = −0.158 *p* = 0.336	r = −0.126 *p* = 0.696	r = 0.109 *p* = 0.688	**r = −0.656** ***p *** **= 0.003**	**r = −0.676** ***p *** **= 0.002**	**r = −0.976** ***p *** **< 0.001**	**r = −0.870** ***p *** **< 0.001**	**r = −0.743** ***p *** **< 0.001**	**r = −0.737** ***p *** **= 0.001**	r = −0.349 *p* = 0.242	**r = −0.657** ***p *** **= 0.015**

Abbreviations: BMD = bone mineral density; DXA = dual-energy X-ray absorptiometry; MOF = major osteoporotic fracture; HF = hip fracture; FRAX1 = MOF without femoral neck BMD; FRAX2 = MOF with femoral neck BMD; FRAXplus1 = MOF without femoral neck BMD adjusted for HPT; FRAXplus2 = MOF with femoral neck BMD adjusted for HPT; FRAXplus3 = MOF adjusted for lumbar BMD; FRAX3 = HF without femoral neck BMD; FRAX4 = HF with femoral neck BMD; FRAXplus4 = HF without femoral neck BMD adjusted for HPT; FRAXplus5 = HF with femoral neck BMD adjusted for HPT; FRAXplus6 = HF adjusted for lumbar BMD; NC-HPT = normocalcemic primary hyperparathyroidism; P1NP = procollagen 1 N-terminal propeptide; SD = standard deviation.

## 4. Discussion

To our best knowledge, this is the first study to address the use of FRAXplus for estimating 10-year osteoporotic fracture probabilities in patients confirmed with HPT, and among the first studies ever to provide an adjustment of current FRAX-based scores, not only for the presence of HPT, but, also, for the values of lumbar BMD at central DXA [35,36,37]. Additionally, a sub-analysis of hypercalcemic and normocalcemic variants was provided, noting the massive interest of the medical community for this new face of HPT (NC-HPT) amid contemporary era [30,38,39,40]. To date, the milder biochemical entity remains an open matter with respect to a standardized diagnosis and management, while the clinical co-ailments might not be mild across lifespan [40,41].

In order to pinpoint an adequate fracture risk estimation, an analysis of the mineral metabolism profile, circulating bone turnover markers, and DXA-based BMDs at four DXA sites was performed, as well. Overall, a cohort of 131 subjects included 51.15% of them with HPT versus age-, menopause duration-, and body mass index-matched (HPT-free) controls. Thus, 64.18% of the individuals with HPT presented HC-HPT, and 35.82% had NC-HPT, both subgroups having a similar age, menopause duration, and body mass index. Approximately 45% of each group (HPT versus non-HPT) suffered from an impaired glucose profile (prediabetes or type 2 diabetes) and 60–70% from arterial hypertension, noting that subjects with non-parathyroid tumors were ruled out, as well as those with genetic forms of HPT and type 1/secondary diabetes in order to eliminate the bias that comes from concurrent (non-PTH-related) hormonal imbalances with negative bone and cardiometabolic effects [42,43,44]. Of note, serum creatinine was found higher in HPT versus controls, but these were intra-normal variations, while the patients with an abnormal renal function/renal hyperparathyroidism were excluded from the start.

As expected, not only serum calcium and PTH, but also serum phosphorus, were statistically significant different, as well as calcium/magnesium ratio in HPT versus (HPT-free) controls. Recently, this ratio gained attention in order to integrate the nutrients intake, as well as the anomalies of the hormones (such as PTH and vitamin D) that could interfere with the mineral status and the bone health [45,46]. A study from 2024 showed that an increased value might serve as an alternative non-invasive biomarker of developing kidney stones in HPT (a cut off 5.24 associates a sensitivity of 73.3% and a specificity of 73%) [46]. In current analysis, this calcium-to-magnesium ratio was higher in HC-HPT versus NC-HPT in addition to higher calcemic levels and PTH, as well as lower serum phosphorus.

An elevated bone turnover in HPT (versus controls) was reflected by increased osteocalcin and CrossLaps, while bone formation marker P1NP did not reach statistical significance. Total alkaline phosphatase was also statistically significantly higher in HPT versus non-HPT. However, the bone fraction of this biomarker/enzyme might have been more accurate [47], but it is not available in our hospital.

In HPT group, total serum calcium and PTH positively correlated with bone resorption marker CrossLaps. On the other hand, HC-HPT subgroup showed a similar profile with NC-HPT with regard to the bone turnover markers. In HC-HPT subgroup, total serum calcium positively correlated with CrossLaps, while in NC-HPT subgroup, serum phosphorus positively associated with osteocalcin and PTH with CrossLaps. These blood assays captured the essence of bone status disturbances in HPT and might serve as predictors of the anti-osteoporotic intervention in HPT and post-parathyroidectomy outcomes [48].

All four DXA sites showed statistically significant lower BMDs and TBS levels in HPT versus non-HPT, with an osteoporosis rate of 50.75% versus 6.25% (*p* < 0.001). In HPT group, total calcium of 10.75 mg/dL predicted osteoporosis with 61.8% sensitivity and 76.7% specificity (ROC analysis), respectively, a PTH value of 100.28 pg/mL (sensitivity of 61.8% and specificity of 72.4%). In HPT group, total serum calcium negatively correlated with lumbar BMD and third distal radius T-score, the same as PTH. TBS was negatively correlated with PTH in HPT group and in HC-HPT subgroup. In NC-HPT subgroup, PTH correlated with radius T-score.

Lumbar, total hip, and third distal radius BMD was statistically significant lower in HC-HPT versus NC-HPT, with a higher osteoporosis prevalence (60.47% versus 33.33%). Osteoporosis remains a hallmark in HPT, parathyroids-released PTH playing a crucial role in calcium/phosphate homeostasis by primarily acting on bone and kidney. The interplay with bone remodeling by stimulating the bone cells (osteoblasts and osteocytes and then osteoclasts) connects the calcemic profile to the bone formation/resorption [49]. Despite a core complication in HPT, osteoporosis might remain under investigated and, thus, undertreated in real-life settings. For instance, a retrospective, single-center study published in 2025 showed that only 45.4% of the parathyroidectomy candidates were pre-operatory evaluated at DXA scan [50].

Notably, the rate of prevalent fractures was similar in HPT versus controls (19.40% vs. 14.06%, *p* = 0.414). A bias might come from the fact that patients who suffered previous fractures might have been treated with specific anti-osteoporotic drugs, a population that we ruled out for this analysis. Interestingly, the rate of fractures was lower in HC-HPT versus NC-HPT (10.87% vs. 33.33%), which may come from the same bias of excluding subjects with pre-treatment, but, also, from the fact that normocalcemic variant might remain underrecognized in individuals with fragility fractures amid a real-life approach. For instance, a prevalence of 0.18% was reported for normocalcemic variant of HPT among consecutive unselected individuals undergoing central DXA scan, suggesting a rather low index of suspicion in the general population [24].

Based on these results, osteoporosis rate suggested that hypercalcemic status in HPT is prone to more bone damage than normocalcemic variant, but the rate of fractures sustained the contrary. Noting this gap in addressing HPT-related osteoporosis/osteoporotic fractures, the fracture risk calculators become emergent tools for the daily practice. In this study, the 10-year fracture risk for MOF and HF was statistically significantly higher in HPT versus controls only for the calculation with femoral neck BMD. FRAXplus showed that for both estimations (MOF and HF) with introduction of lumbar BMD remained higher than controls (4.55% vs. 3.7%, *p* = 0.004, respectively, 1.05% vs. 0.5%, *p* = 0.002). The similar values between FRAX-based probabilities without the use of femoral neck BMD (HPT versus non-HPT controls) suggested it might not be so useful in HPT population, thus the need of novel models of risk estimation [33,51,52,53].

In HPT group, both 10-year fracture risk for MOF and HF were higher if adjustment for HPT was applied. The highest 10-year fracture risk for MOF was obtained for HPT adjustment with femoral neck BMD (5.9%) versus the estimation without using femoral neck BMD (5.25%, *p* = 0.001), respectively, versus the probability with adjustment for lumbar BMD (4.55%, *p* < 0.001). The same observation was available for HF: 1.4% versus 1.2% (*p* = 0.028), respectively, versus 1.05% (*p* < 0.001). Moreover, PTH positively correlated with 10-year hip fracture risk with HPT adjustment, calculated without femoral neck BMD (FRAXplus 4: r = 0.257, *p* = 0.049), and P1NP inversely associated with the 10-year hip fracture risk without femoral neck BMD (FRAX3: r = −0.407, *p* = 0.049). P1NP negatively correlated with 10-year fracture risk for MOF without femoral neck BMD (FRAX1: r = −0.416, *p* = 0.043) and with the estimation including HPT adjustment (FRAXplus1: r = −0.404, *p* = 0.05).

All 10-year probabilities based on FRAX and FRAXplus models showed similar values in HC-HPT versus NC-HPT, which implies that current algorithms might not make a clear distinction between the subtype of hyperparathyroidism, according to these primary data. On the other hand, the mentioned statistically significant results with respect to FRAXplus-based estimations for MOF/HF were found within each of these subgroups, as well, confirming their usefulness regardless of the hyperparathyroidism variant of presentation.

Limitations of the current study involve the sample size and the retrospective design. Considering the retrospective single-center design, no prior sample size calculation was performed. Additionally, given the number of statistical comparisons performed, a risk of type I error cannot be excluded; therefore, the results should be considered descriptive-analytical and warrant validation in larger prospective studies.

In addition, although the exploratory nature of the study is acknowledged, a limited sensitivity analysis or simplified multivariable model adjusting for a small number of clinically relevant covariates (e.g., age, dyslipidaemia, creatinine, and prior fractures) would further strengthen the robustness of these findings and further extension in other population, as shown by FRAX application all over the world might encapsulate its optimum use [54,55,56]. Given the relatively small NC-HPT subgroup and the large number of comparisons performed, the subgroup findings should continue to be interpreted cautiously and framed primarily as hypothesis-generating observations [55,57,58].

Also, we restricted the application of novel FRAX to menopausal women. Future prospective, longitudinal studies might include other populations (e.g., males, premenopausal women, hereditary HPT, renal hyperparathyroidism, etc.). Also, the interventional cutoff for practical intervention starting from FRAX models is yet to be established [57,58,59].

Moreover, no relationship with anti-interventional strategy following the osteoporosis detection (specific medication to reduce the fracture risk versus post-parathyroidectomy improvement) was analyzed, noting that parathyroid tumor removal is expected to improve the fracture risk by correcting PTH level and even reducing the pro-inflammatory status and potentially the metabolic disturbances such as the glucose profile [60,61,62,63].

Potential confounders might involve ddyslipidaemia, creatinine levels, bone turnover markers, comorbidities, and prior fracture history.

In addition, the ROC-based cutoffs should be described as exploratory only at this point. The clinical applicability without **external** validation is yet to be determined.

Of note, the higher prevalence of fragility fractures in NC-HPT (despite lower osteoporosis rates) is intriguing, and possible explanations include selection bias, exclusion of treated osteoporosis patients, and underdiagnosis differences.

Further expansion of the current research might include multimodal algorithms to estimate, not only the fracture risk, but also the risk of other comorbidities in HPT, by integrating the clinical panel, biochemical, and hormonal assessments (e.g., 25-hydroxyvitamin D and bone turnover markers), and imaging findings (e.g., parathyroid scintigram). Simultaneous TBS integration in fracture assessment might help, as well as machine learning-based radiomics analyses of spine CT images [56,55].

## 5. Conclusions

To our best knowledge, this is the first study to address the use of FRAXplus in HPT. The similar values between FRAX-based probabilities without the use of femoral neck BMD in HPT versus non-HPT controls suggested that this traditional estimation might not be so useful in HPT population, thus the need of novel models such as HPT adjustment according to novel algorithm. HPT adjustment (FRAXplus) provided a higher MOF/HF risk versus non-adjustment (FRAX). All 10-year probabilities based on FRAX and FRAXplus models showed similar values in HC-HPT versus NC-HPT, which implies that current algorithms might not make a clear distinction between HPT subtypes, yet the statistically significant results within each of these subgroups sustain the FRAXplus application regardless of the variant.

## Figures and Tables

**Table 7:** Sensitivity and specificity for the cutoff value of 100.28 pg/mL of parathormone for predicting osteoporosis among patients in group HPT.

Parameter	Cutoff Value for Predicting Osteoporosis (pg/mL)	AUC	Sensitivity (%)	Specificity (%)	Youden Index
**Parathormone**	100.28	0.659	61.80	72.40	0.342

Abbreviations: AUC = area under the curve; HPT = hyperparathyroidism.

## Data Availability

All available data are in the article.

## Supplementary Materials

The following supporting information can be downloaded at: https://www.mdpi.com/article/10.3390/life16060932/s1.

## Abbreviations

The following abbreviations are used in this manuscript:

AUC	area under the curve
BMD	bone mineral density
DXA	dual-energy X-ray absorptiometry
FRAX	Fracture Risk Assessment Tool
HPT	primary hyperparathyroidism
HC-HPT	hypercalcemic primary hyperparathyroidism
HF	hip fracture
MOF	major osteoporotic fractures
NC-HPT	normocalcemic primary hyperparathyroidism
N	number of patients
PTH	parathormone
P1NP	procollagen 1 N-terminal propeptide
ROC	receiver operating characteristic
Q	quartile
SD	standard deviation
TBS	trabecular bone score
