# Supplementary material for: Future Directions in Hypercalcemic and Normocalcemic Primary Hyperparathyroidism: FRAXplus for 10-Year Fracture Risk Assessment (A Retrospective Study)
Source: Life (Basel). 2026 Jun 1;16(6):932. doi: 10.3390/life16060932 (PMC13301923; doi:10.3390/life16060932)
Supplement: Supplementary file 1 [file life-16-00932-s001.zip › life-4288146-supplementary.pdf]

This is FRAX/FRAXplus use (study population on point):

Firstly, this is standard FRAX:

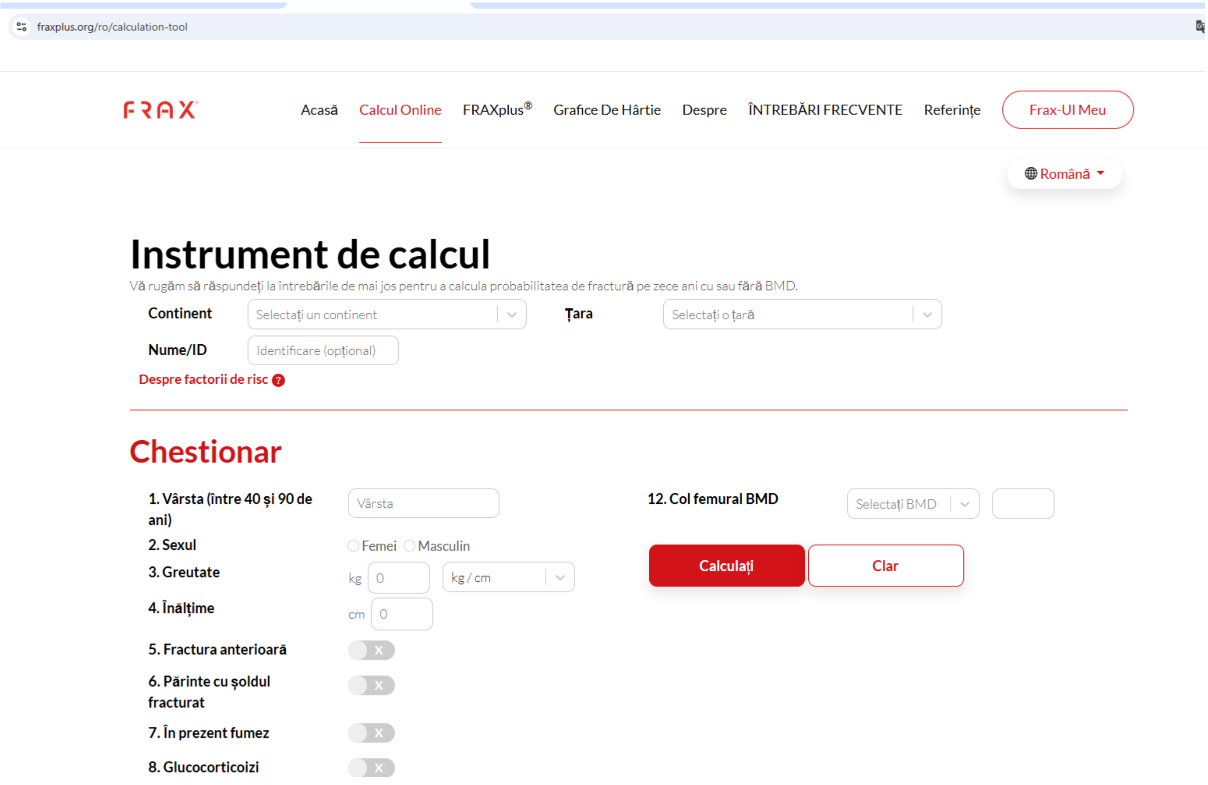

Standard inputs are as follows:

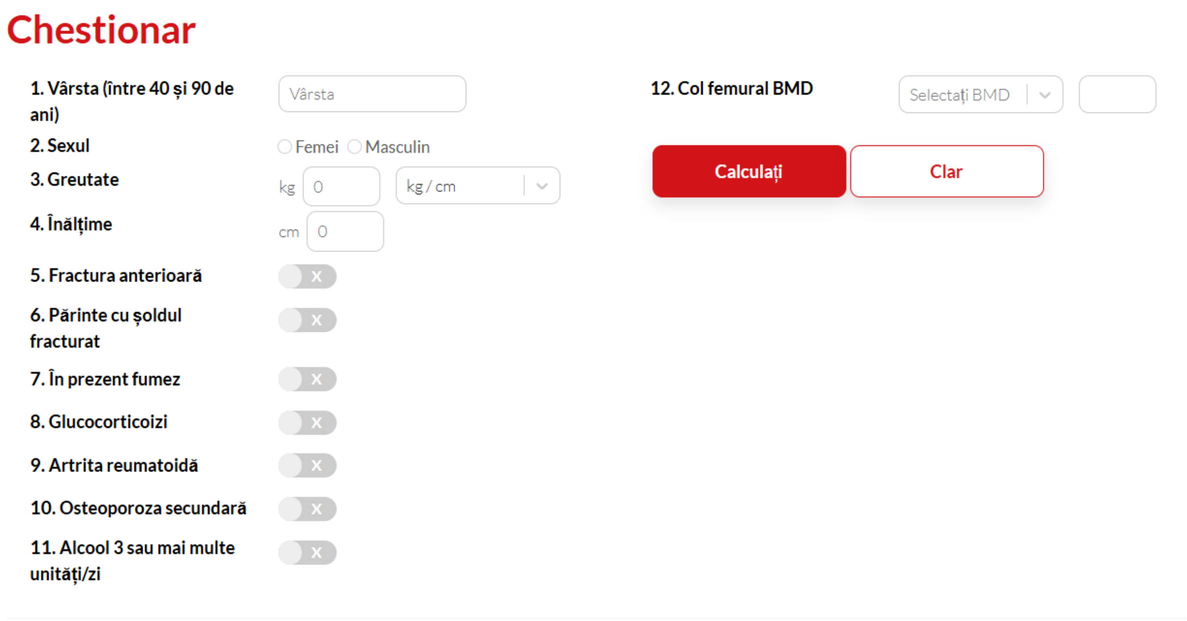

For FRAX calculation in one patient, femoral neck BMD may be used or not:

12. Col femural BMD

Selectați BMD

Calculați

Clar

FRAXplus

Fur further using FRAXplus, this input (femoral neck BMD) in mandatory

12. Col femural BMD

Selectați BMD

Calculați

Clar

fraxplus.org/ro/calculation-tool

FRAX

Acasă Calcul Online FRAXplus®

The panel of potential adjustments for FRAXplus (for one individual, an adjustment should be done one by one, not combined elements)

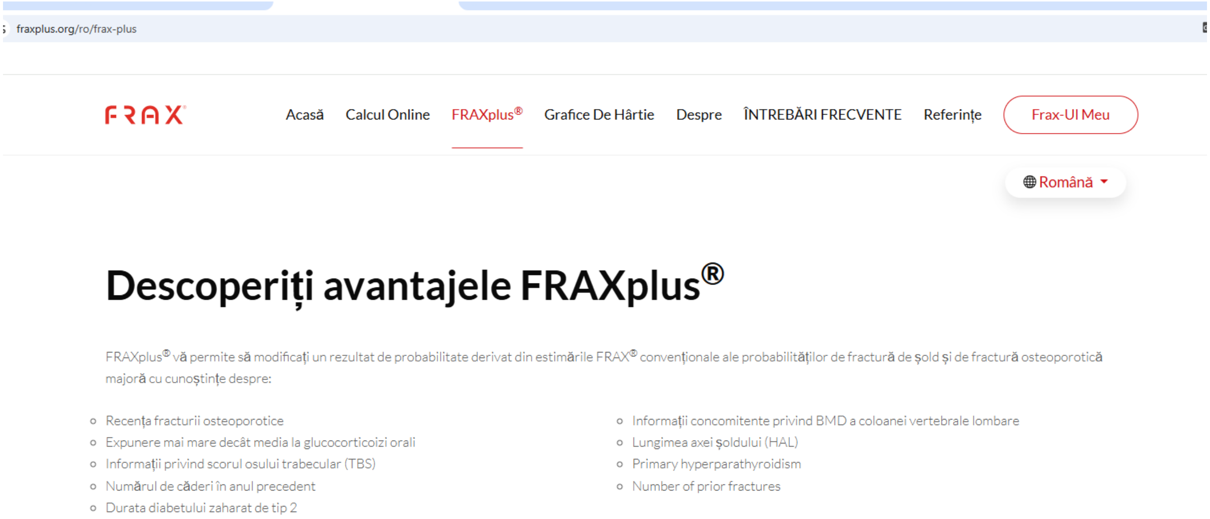
fraxplus.org/ro/frax-plus

FRAX | Acasă | Calcul Online | FRAXplus® | Grafice De Hârtie | Despre | ÎNTREBĂRI FRECVENTE | Referințe | Frax-UI Meu

Română

Descoperiți avantajele FRAXplus®

FRAXplus® vă permite să modificați un rezultat de probabilitate derivat din estimările FRAX® convenționale ale probabilităților de fractură de șold și de fractură osteoporotică majoră cu cunoștințe despre:

- Recența fracturii osteoporotice
- Expunere mai mare decât media la glucocorticoizi orali
- Informații privind scorul osului trabecular (TBS)
- Numărul de căderi în anul precedent
- Durata diabetului zaharat de tip 2
- Informații concomitente privind BMD a coloanei vertebrale lombare
- Lungimea axei șoldului (HAL)
- Primary hyperparathyroidism
- Number of prior fractures

For instance, adjustments for lumbar BMD is currently applicable

- Informații concomitente privind BMD a coloanei vertebrale lombare

Or adjustment for primary hyperparathyroidism

- Primary hyperparathyroidism
